# Forbush decreases: Algorithm generated dataset

**DOI:** 10.1016/j.dib.2020.106463

**Published:** 2020-10-28

**Authors:** O. Okike

**Affiliations:** Department of Industrial Physics, Faculty of Science, Ebonyi State University, Abakaliki 480, Nigeria

**Keywords:** Forbush decreases, Fourier transform, Algorithm, Cosmic ray anisotropy, Simultaneity, Forbush event identification

## Abstract

Efforts to correctly detect Forbush decreases (FDs) in the cosmic ray (CR) intensity flux are ongoing (see [2], [7]). The FD data presented here are a part of the data generated in [6] in a recent investigation of the effects of CR anisotropies on the simultaneity of FDs. A part of the simultaneous and non-simultaneous FDs detected from both raw and Fourier transformed CR data are presented. Some of the filtered CR anisotropies are also presented. The datasets are interesting as they provide an opportunity to investigate the interaction of CR anisotropies on FDs. [6] identified FDs from both raw and Fourier transformed CR data. For the FDs identified in the raw data, the impact of diurnal anisopropy was not removed. Additionally, FDs were identified using a careful adjustment for diurnal anisotropy. The results of FD catalogues for ten CR stations are presented. All are calculated with the same method and selection criteria. Thus these catalogues can be united contrary to the indication of [1], allowing comparison of Chree, regression or correction analysis based on CR data from isolated neutron monitors [9]. These data can also be used in the analysis of FD event simultaneity. Several claims on FD event simultaneity by some authors ([5], [8]) using few FDs can be verified using the data. The diagrams show the relationships between raw and Fourier transformed CR data as well as CR anisotropic variations at different locations. The similarities and differences between these figures could be used to discuss the impact of rigidity on diurnal anisotropy and FDs. The quantitative amplitudes of the diurnal anisotropy calculated at the time of FDs could be useful in such investigation. Okike [6] calculated the amplitude of diurnal anisotropies accompanying FDs and are presented here. The large number of strong and small FDs selected from each station for the year 2003 may encourage reanalysis of previous work employing only 3 or 4 FDs in each year. Many studies relating FDs to terrestrial effects have focused on a single FD without strong evidence that it is generalizable to others. This may attract criticism that the conclusions drawn do not extend to all FDs. The diagrams presented here are similar to those presented in [6] for the Climax station. Rather than display separate FD events, the complete intensity variation together with the accompanying FDs are clearly displayed. Solar wind plasma data may be displayed alongside the data for comparative purposes. It is hoped that this approach will answer the numerous objections made to solar-terrestrial analysis involving FDs.

## Specifications Table

**Table:** 

Subject	Atmospheric Science.
Specific subject area	This is an aspect of space physics that examines the impact of solar and extra-galactic emissions on the Earth’s weather and human civilization. Arguably, several health hazards have been attributed to the influence of incoming energetic particles accelerated to very high speeds by the interplanetary magnetic field. Those particles are commonly called cosmic rays (CRs). The invention of neutron monitors allowed continuous recording of these CRs. Rapid fluctuations in CR flux are considered to be an effect of energetic particle solar emissions arising from solar flares, coronal holes or coronal mass ejections (CMEs). While short-term rapid depressions in the CR intensity are described as Forbush decreases, transient increases are referred to as ground level enhancements (GLEs). Forbush decreases, which are the focus of this work, is considered the most important CR intensity variation ([10], [12]).
Type of data	Table
	Figure
How data were acquired	Raw cosmic ray data were downloaded from the website (http://cr0.izmiran.ru/common) created by the Pushkov Institute of Terrestrial Magnetism, Ionosphere, and Radio Wave Propagation, Russian Academy of Sciences (IZMIRAN). All analyses were performed using the R Language for Statistics and Graphics [11]. The resulting FD characteristics (event magnitude and time) are presented in the diagrams and Tables below.
Data format	Analyzed: 2003.05.19 00:00 6970 2003.05.20 00:00 6961 2003.05.21 00:00 6961 2003.05.22 00:00 6932 2003.05.23 00:00 6973 2003.05.24 00:00 6988 2003.05.25 00:00 6954 2003.05.26 00:00 6903 Filtered: 2003-05-02 -2.70584539738971 2003-05-06 -3.15243951516567 2003-05-09 -2.77524440356152 2003-05-14 -0.349316750812932 2003-05-30 -11.9197344086189 2003-06-23 -4.31787003941969 2003-06-27 -0.659678647445929 2003-07-15 -0.980504014083351 2003-07-17 -1.24167329768645 2003-07-24 -0.527673315904678
Parameters for data collection	Computer, electricity, and internet source.
Description of data collection	The raw data (see Supplementary data for the ten CR stations were first downloaded from the IZMIRAN website (http://cr0.izmiran.ru/common). The raw data were processed before analysis as follows. The date/time format of the raw data was ”YYYY.mm.dd HH:MM”, followed by the CR count. This was transformed into ”dd mm yy” format followed by the CR count. Missing counts, recorded as zeros, were changed to NA (data Not Available). These transformations were performed in ”awk”, a text processing utility usually available on UNIX operating systems.
Data source location	Neutron monitors are located at various locations around the world. However, the IZMIRAN group collects data from all these stations and stores them in same format. The geographical locations of the stations are included in the website.
Data accessibility	Data are available with this article.
Related research article	O. Okike Automated Detection of Simultaneous/Non-Simultaneous Forbush Decreases and the Associated Cosmic Ray Phenomena Journal of Atmospheric and Solar-Terrestrial Physics, (2020), 211, 105460. https://doi.org/10.1016/j.jastp.2020.105460

## Value of the Data

• Although the potential for bias in studying diurnal anisotropy using isolated neutron monitor data has long been known, it has not been quantified. The two datasets provide an opportunity for comparative studies of the interaction of diurnal anisotropy with Forbush events.
• The data presented here may be useful to a number of researchers, including those analyzing the relation between FDs and their solar sources, the impact of CRs on terrestrial weather or the global simultaneity of FDs.
• We have presented the data in such a manner that almost every CR investigator can employ it directly in analysis. The event magnitude and date are presented and can be used for either superposition or correlation analysis.
• The quantitative relation between FD and the amplitude of the CR anisotropy are of interest to some researchers, e.g. [3]. The magnitude of FDs and the usual/abnormal amplitude presented in Table 9, Table 10, Table 11, Table 12, Table 13, Table 14, Table 15, Table 16, Table 17, Table 18 could be used to validate the result of the global survey method [4] used by the Russian group to calculate the amplitude of anisotropies associated with FDs.

**Table 1:** Catalogues of Forbush Decreases Identified from Preliminary Processed CR Data at APTY, MCMD, MOSC, OULU and SNAE CR stations.

	Date	APTY	Date	MCMD	Date	MOSC	Date	OULU	Date	SNAE
1	2003-01-03	−0.28	2003-01-04	−1.74	2003−01−04	−1.16	2003-01-04	−1.37	2003-01-04	−1.97
2	2003-01-24	−3.00	2003-01-07	−0.29	2003-01-06	−0.84	2003-01-11	−0.70	2003-01-10	−1.98
3	2003-01-27	−4.94	2003-01-10	−0.97	2003-01-11	−1.56	2003-01-24	−2.48	2003-01-24	−2.04
4	2003-02-03	−4.93	2003-01-25	−2.08	2003-01-24	−3.03	2003-01-27	−4.57	2003-01-27	−4.22
5	2003-02-18	−5.52	2003-01-27	−5.07	2003-01-27	−5.13	2003-02-02	−4.64	2003-02-02	−4.31
6	2003-03-20	−4.33	2003-01-30	−1.53	2003-02-03	−2.29	2003-02-13	−0.42	2003-02-15	−1.59
7	2003-03-31	−3.18	2003-02-02	−4.61	2003-02-18	−4.20	2003-02-18	−4.59	2003-02-19	−4.80
8	2003-04-05	−0.31	2003-02-19	−4.35	2003-02-21	−3.37	2003-03-07	−0.21	2003-03-16	−0.78
9	2003-04-10	−4.78	2003-03-04	−0.32	2003-03-05	−0.93	2003-03-10	−1.16	2003-03-20	−2.60
10	2003-05-02	−2.71	2003-03-16	−0.76	2003-03-10	−0.88	2003-03-17	−1.18	2003-03-31	−4.05
11	2003-05-06	−3.15	2003-03-20	−3.13	2003-03-15	−1.18	2003-03-20	−2.51	2003-04-11	−4.64
12	2003-05-09	−2.78	2003-03-31	−2.44	2003-03-20	−2.52	2003-03-31	−2.56	2003-04-27	−0.85
13	2003-05-14	−0.35	2003-04-11	−3.90	2003-04-01	−1.98	2003-04-11	−3.76	2003-05-03	−2.17
14	2003-05-30	−11.92	2003-04-14	−2.34	2003-04-11	−5.24	2003-05-02	−2.19	2003-05-06	−0.67
15	2003-06-23	−4.32	2003-04-22	−0.66	2003-04-14	−2.89	2003-05-06	−2.55	2003-05-09	−2.27
16	2003-06-27	−0.66	2003-05-02	−1.31	2003-04-30	−0.76	2003-05-09	−2.93	2003-05-31	−12.83
17	2003-07-15	−0.98	2003-05-06	−1.58	2003-05-02	−0.75	2003-05-14	−0.42	2003-06-23	−4.23
18	2003-07-17	−1.24	2003-05-09	−2.83	2003-05-07	−1.36	2003-05-30	−11.79	2003-06-28	−0.47
19	2003-07-24	−0.53	2003-05-31	−11.87	2003-05-09	−2.21	2003-06-23	−4.29	2003-07-15	−0.43
20	2003-07-27	−2.24	2003-06-23	−4.36	2003-05-31	−11.66	2003-06-27	−0.90	2003-07-21	−0.25
21	2003-07-30	−2.52	2003-06-27	−0.74	2003-06-24	−4.51	2003-07-03	−0.35	2003-07-27	−6.01
22	2003-08-05	−0.25	2003-07-14	−0.53	2003-06-27	−0.88	2003-07-15	−2.04	2003-08-02	−2.00
23	2003-08-10	−0.14	2003-07-17	−0.00	2003-07-15	−1.74	2003-07-17	−1.31	2003-08-05	−1.62
24	2003-08-18	−1.57	2003-07-20	−0.90	2003-07-17	−0.78	2003-07-22	−0.13	2003-08-11	−0.53
25	2003-09-06	−0.09	2003-07-27	−3.45	2003-07-22	−0.48	2003-07-27	−1.28	2003-08-19	−3.46
26	2003-09-10	−1.51	2003-07-31	−2.42	2003-07-27	−2.44	2003-07-30	−1.13	2003-09-07	−0.10
27	2003-09-12	−1.71	2003-08-02	−1.60	2003-07-30	−2.07	2003-08-02	−0.73	2003-09-09	−2.26
28	2003-09-14	−2.34	2003-08-05	−1.89	2003-08-01	−2.77	2003-08-04	−0.17	2003-09-12	−1.57
29	2003-09-18	−3.81	2003-08-08	−0.23	2003-08-05	−1.03	2003-09-10	−0.12	2003-09-17	−3.30
30	2003-09-22	−2.63	2003-08-10	−0.41	2003-08-09	−0.60	2003-09-12	−0.72	2003-09-21	−3.21
31	2003-09-30	−0.34	2003-08-12	−0.33	2003-08-18	−1.78	2003-09-14	−1.30	2003-09-27	−1.51
32	2003-10-08	−1.87	2003-08-18	−1.12	2003-08-30	−0.56	2003-09-18	−3.12	2003-09-30	−1.87
33	2003-10-25	−0.57	2003-09-04	−1.53	2003-09-14	−2.34	2003-09-20	−2.29	2003-10-07	−2.29
34	2003-10-31	−29.26	2003-09-10	−1.97	2003-09-18	−3.32	2003-09-25	−0.29	2003-10-31	−33.70
35	2003-11-07	−1.87	2003-09-17	−4.16	2003-09-21	−3.29	2003-09-27	−0.04	2003-11-07	−0.51
36	2003-11-17	−0.13	2003-09-21	−2.44	2003-09-25	−1.76	2003-09-30	−0.89	2003-11-18	−0.21
37	2003-11-24	−3.36	2003-09-25	−1.51	2003-09-30	−1.52	2003-10-07	−3.02	2003-11-23	−4.81
38	2003-12-08	−0.63	2003-09-30	−0.70	2003-10-06	−0.20	2003-10-25	−0.49	2003-12-11	−2.52
39	2003-12-11	−4.18	2003-10-07	−2.31	2003-10-09	−1.28	2003-10-31	−28.32	2003-12-23	−0.89
40	2003-12-15	−2.40	2003-10-25	−1.58	2003-10-31	−27.67	2003-11-07	−0.69	2003-12-25	−1.21
41	2003-12-19	−2.16	2003-10-31	−31.25	2003-11-07	−2.63	2003-11-24	−4.19	2003-12-29	−1.66
42	2003-12-23	−2.33	2003-11-07	−2.00	2003-11-24	−4.32	2003-12-04	−0.08		
43	2003-12-25	−3.53	2003-11-18	−1.12	2003-12-01	−0.48	2003-12-08	−0.91		
44	2003-12-28	−2.26	2003-11-21	−2.02	2003-12-05	−0.42	2003-12-10	−3.05		
45			2003-11-23	−3.26	2003-12-11	−3.29	2003-12-14	−1.44		
46			2003-12-01	−0.05	2003-12-23	−1.65	2003-12-24	−1.38		
47			2003-12-10	−1.66	2003-12-28	−0.01	2003-12-28	−0.08		
48			2003-12-12	−1.68						
49			2003-12-15	−1.15						
50			2003-12-22	−1.54						
51			2003-12-28	−0.35

**Table 2:** Catalogues of simultaneous FDs at APTY and OULU, APTY and SNAE and MCMD and SNAE.

	Date	APTY	OULU	Date	APTY	SNAE	Date	MCMD	SNAE
1	2003-01-24	−3.00	−2.48	2003-01-24	−3.00	−2.04	2003-01-04	−1.74	−1.97
2	2003-01-27	−4.94	−4.57	2003-01-27	−4.94	−4.22	2003-01-10	−0.97	−1.98
3	2003-02-18	−5.52	−4.59	2003-03-20	−4.33	−2.60	2003-01-27	−5.07	−4.22
4	2003-03-20	−4.33	−2.51	2003-03-31	−3.18	−4.05	2003-02-02	−4.61	−4.31
5	2003-03-31	−3.18	−2.56	2003-05-06	−3.15	−0.67	2003-02-19	−4.35	−4.80
6	2003-05-02	−2.71	−2.19	2003-05-09	−2.78	−2.27	2003-03-16	−0.76	−0.78
7	2003-05-06	−3.15	−2.55	2003-06-23	−4.32	−4.23	2003-03-20	−3.13	−2.60
8	2003-05-09	−2.78	−2.93	2003-07-15	−0.98	−0.43	2003-03-31	−2.44	−4.05
9	2003-05-14	−0.35	−0.42	2003-07-27	−2.24	−6.01	2003-04-11	−3.90	−4.64
10	2003-05-30	−11.92	−11.79	2003-08-05	−0.25	−1.62	2003-05-06	−1.58	−0.67
11	2003-06-23	−4.32	−4.29	2003-09-12	−1.71	−1.57	2003-05-09	−2.83	−2.27
12	2003-06-27	−0.66	−0.90	2003-09-30	−0.34	−1.87	2003-05-31	−11.87	−12.83
13	2003-07-15	−0.98	−2.04	2003-10-31	−29.26	−33.70	2003-06-23	−4.36	−4.23
14	2003-07-17	−1.24	−1.31	2003-11-07	−1.87	−0.51	2003-07-27	−3.45	−6.01
15	2003-07-27	−2.24	−1.28	2003-12-11	−4.18	−2.52	2003-08-02	−1.60	−2.00
16	2003-07-30	−2.52	−1.13	2003-12-23	−2.33	−0.89	2003-08-05	−1.89	−1.62
17	2003-09-10	−1.51	−0.12	2003-12-25	−3.53	−1.21	2003-09-17	−4.16	−3.30
18	2003-09-12	−1.71	−0.72				2003-09-21	−2.44	−3.21
19	2003-09-14	−2.34	−1.30				2003-09-30	−0.70	−1.87
20	2003-09-18	−3.81	−3.12				2003-10-07	−2.31	−2.29
21	2003-09-30	−0.34	−0.89				2003-10-31	−31.25	−33.70
22	2003-10-25	−0.57	−0.49				2003-11-07	−2.00	−0.51
23	2003-10-31	−29.26	−28.32				2003-11-18	−1.12	−0.21
24	2003-11-07	−1.87	−0.69				2003-11-23	−3.26	−4.81
25	2003-11-24	−3.36	−4.19						
26	2003-12-08	−0.63	−0.91						
27	2003-12-28	−2.26	−0.08

**Table 3:** Catalogues of non-simultaneous FDs at APTY and OULU stations.

	Date	APTY	Date	OULU
1	2003-01-03	−0.28	2003-01-04	−1.37
2	2003-02-03	−4.93	2003-01-11	−0.70
3	2003-04-05	−0.31	2003-02-02	−4.64
4	2003-04-10	−4.78	2003-02-13	−0.42
5	2003-07-24	−0.53	2003-03-07	−0.21
6	2003-08-05	−0.25	2003-03-10	−1.16
7	2003-08-10	−0.14	2003-03-17	−1.18
8	2003-08-18	−1.57	2003-04-11	−3.76
9	2003-09-06	−0.09	2003-07-03	−0.35
10	2003-09-22	−2.63	2003-07-22	−0.13
11	2003-10-08	−1.87	2003-08-02	−0.73
12	2003-11-17	−0.13	2003-08-04	−0.17
13	2003-12-11	−4.18	2003-09-20	−2.29
14	2003-12-15	−2.40	2003-09-25	−0.29
15	2003-12-19	−2.16	2003-09-27	−0.04
16	2003-12-23	−2.33	2003-10-07	−3.02
17	2003-12-25	−3.53	2003-12-04	−0.08
18			2003-12-10	−3.05
19			2003-12-14	−1.44
20			2003-12-24	−1.38

## 1 Data Description

All the processed/filtered data presented in the diagrams and tables in this article are generated from the raw CR data (see Supplementary data). The raw data for each of the ten CR stations are downloaded from http://cr0.izmiran.ru/common. These raw CR daily averages are presented at the IZMIRAN website (http://cr0.izmiran.ru/common) using the American Standard Code for Information Interchange (ASCII) format. The data format for each of the raw data from the ten stations are the same (YYYY.mm.dd HH:MM, followed by the CR count).

Each file in the supplementary material represents raw data from one station. The files are indicated by the stations’ four lettered abbreviated names (see table 1 of [6]). Raw data for Climax CR station, for example, appears as CLMX whereas SOPO stands for raw CR data from South Polo NM. Some of the relevant characteristics of these stations such as latitude, longitude, rigidity and altitude are reported in table 1 of [6]. The full and abbreviated names for each of the ten stations are also included in the table.

## 2 Catalogues of Forbush Decreases Identified from Preliminary Processed CR Data

These are displayed in Table 1, Table 2, Table 3, Table 4, Table 5, Table 6. The CR station names and abbreviations are Apatity (APTY), Sanae (SNAE), Moscow (MOSC), Oulu (OULU), Magadan (MGDN), Potchefstroom, (PTFM) and South Pole (SOPO), McMurdo (MCMD) and Inuvick (INVK). Simultaneous FDs are presented in Table 3 whereas non-simultaneous FDs are in Tables 4 and 5. Simultaneous FDs at APTY, MCMD, MOSC, OULU and SNAE are presented in Table 6.

**Table 4:** Catalogues of non-simultaneous FDs at APTY and SNAE stations.

	Date	APTY	Date	SNAE
1	2003-01-03	−0.28	2003-01-04	−1.97
2	2003-02-03	−4.93	2003-01-10	−1.98
3	2003-02-18	−5.52	2003-02-02	−4.31
4	2003-04-05	−0.31	2003-02-15	−1.59
5	2003-04-10	−4.78	2003-02-19	−4.80
6	2003-05-02	−2.71	2003-03-16	−0.78
7	2003-05-14	−0.35	2003-04-11	−4.64
8	2003-05-30	−11.92	2003-04-27	−0.85
9	2003-06-27	−0.66	2003-05-03	−2.17
10	2003-07-17	−1.24	2003-05-31	−12.83
11	2003-07-24	−0.53	2003-06-28	−0.47
12	2003-07-30	−2.52	2003-07-21	−0.25
13	2003-08-10	−0.14	2003-08-02	−2.00
14	2003-08-18	−1.57	2003-08-11	−0.53
15	2003-09-06	−0.09	2003-08-19	−3.46
16	2003-09-10	−1.51	2003-09-07	−0.10
17	2003-09-14	−2.34	2003-09-09	−2.26
18	2003-09-18	−3.81	2003-09-17	−3.30
19	2003-09-22	−2.63	2003-09-21	−3.21
20	2003-10-08	−1.87	2003-09-27	−1.51
21	2003-10-25	−0.57	2003-10-07	−2.29
22	2003-11-17	−0.13	2003-11-18	−0.21
23	2003-11-24	−3.36	2003-11-23	−4.81
24	2003-12-08	−0.63	2003-12-29	−1.66
25	2003-12-15	−2.40		
26	2003-12-19	−2.16		
27	2003-12-28	−2.26

**Table 5:** Catalogues of non-simultaneous FDs at MCMD and SNAE stations.

	Date	MCMD	Date	SNAE
1	2003-01-07	−0.29	2003-01-04	−1.97
2	2003-01-25	−2.08	2003-01-10	−1.98
3	2003-01-30	−1.53	2003-02-02	−4.31
4	2003-03-04	−0.32	2003-02-15	−1.59
5	2003-04-14	−2.34	2003-02-19	−4.80
6	2003-04-22	−0.66	2003-03-16	−0.78
7	2003-05-02	−1.31	2003-04-11	−4.64
8	2003-06-27	−0.74	2003-04-27	−0.85
9	2003-07-14	−0.53	2003-05-03	−2.17
10	2003-07-17	−0.002	2003-05-31	−12.83
11	2003-07-20	−0.90	2003-06-28	−0.47
12	2003-07-31	−2.42	2003-07-21	−0.25
13	2003-08-08	−0.23	2003-08-02	−2.00
14	2003-08-10	−0.41	2003-08-11	−0.53
15	2003-08-12	−0.33	2003-08-19	−3.46
16	2003-08-18	−1.12	2003-09-07	−0.10
17	2003-09-04	−1.53	2003-09-09	−2.26
18	2003-09-10	−1.97	2003-09-17	−3.30
19	2003-09-25	−1.51	2003-09-21	−3.21
20	2003-10-25	−1.58	2003-09-27	−1.51
21	2003-11-21	−2.02	2003-10-07	−2.29
22	2003-12-01	−0.05	2003-11-18	−0.21
23	2003-12-10	−1.66	2003-11-23	−4.81
24	2003-12-12	−1.68	2003-12-29	−1.66
25	2003-12-15	−1.15		
26	2003-12-22	−1.54		
27	2003-12-28	−0.35

**Table 6:** Catalogues of simultaneous FDs at APTY, MCMD, MOSC, OULU and SNAE stations.

	Date	APTY	MCMD	MOSC	OULU	SNAE
1	2003-01-27	−4.94	−5.07	−5.13	−4.57	−4.22
2	2003-03-20	−4.33	−3.13	−2.52	−2.51	−2.60
3	2003-05-09	−2.78	−2.83	−2.21	−2.93	−2.27
4	2003-07-27	−2.24	−3.45	−2.44	−1.28	−6.01
5	2003-09-30	−0.34	−0.70	−1.52	−0.89	−1.87
6	2003-10-31	−29.26	−31.25	−27.67	−28.32	−33.70
7	2003-11-07	−1.87	−2.00	−2.63	−0.69	−0.51

**Fig. 1:**
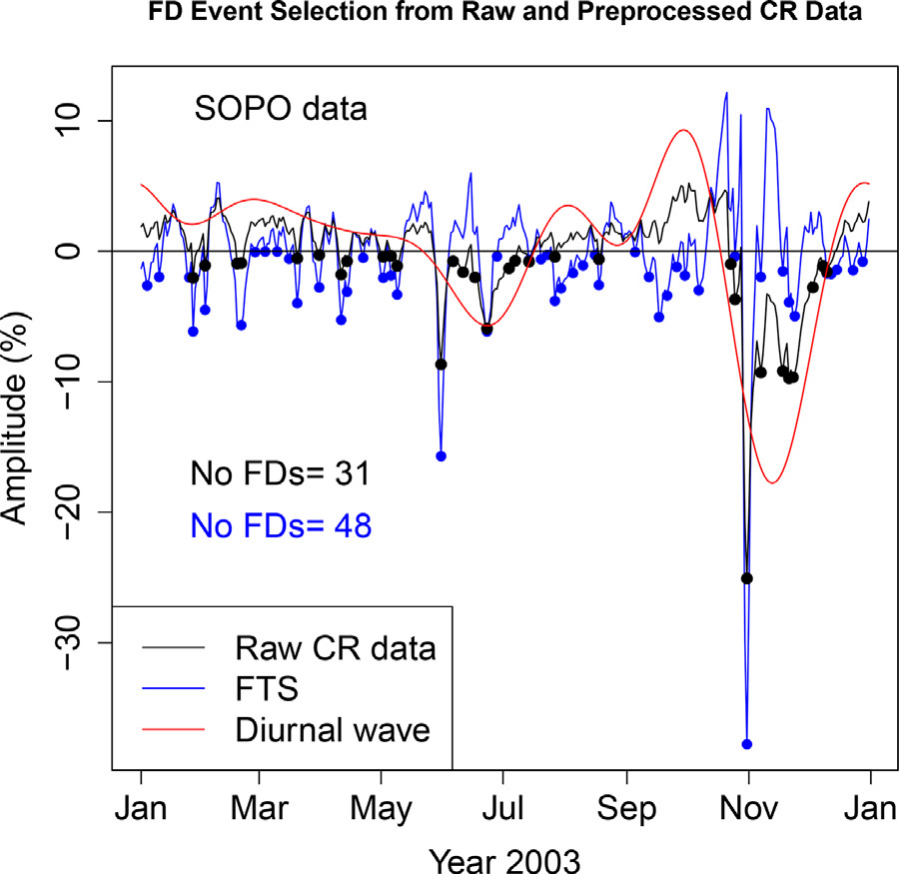
Comparison FDs selected from raw and preliminary processed SOPO CR data. The filled circles are indications of FDs.

**Fig. 2:**
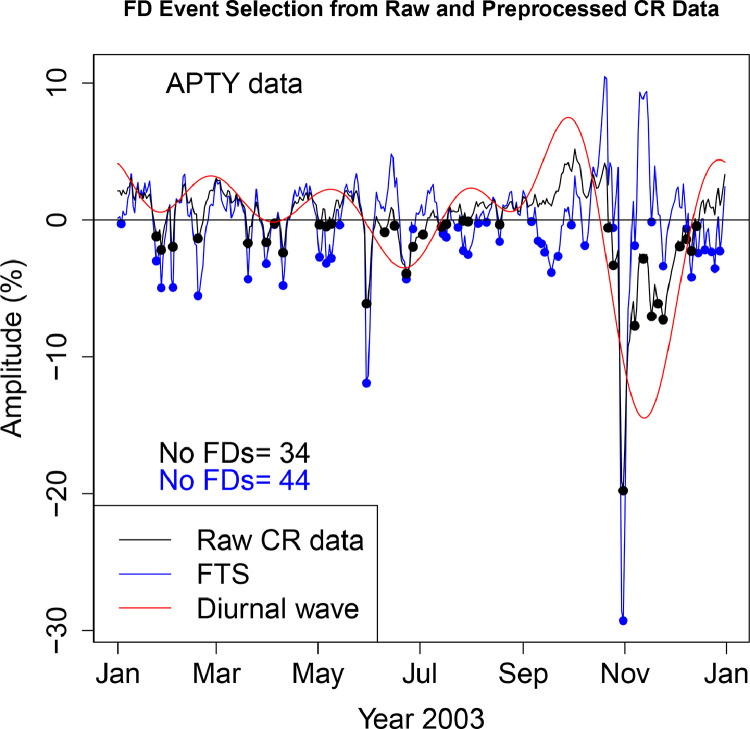
Comparison FDs selected from raw and preliminary processed APTY CR data. The filled circles are indications of FDs.

**Fig. 3:**
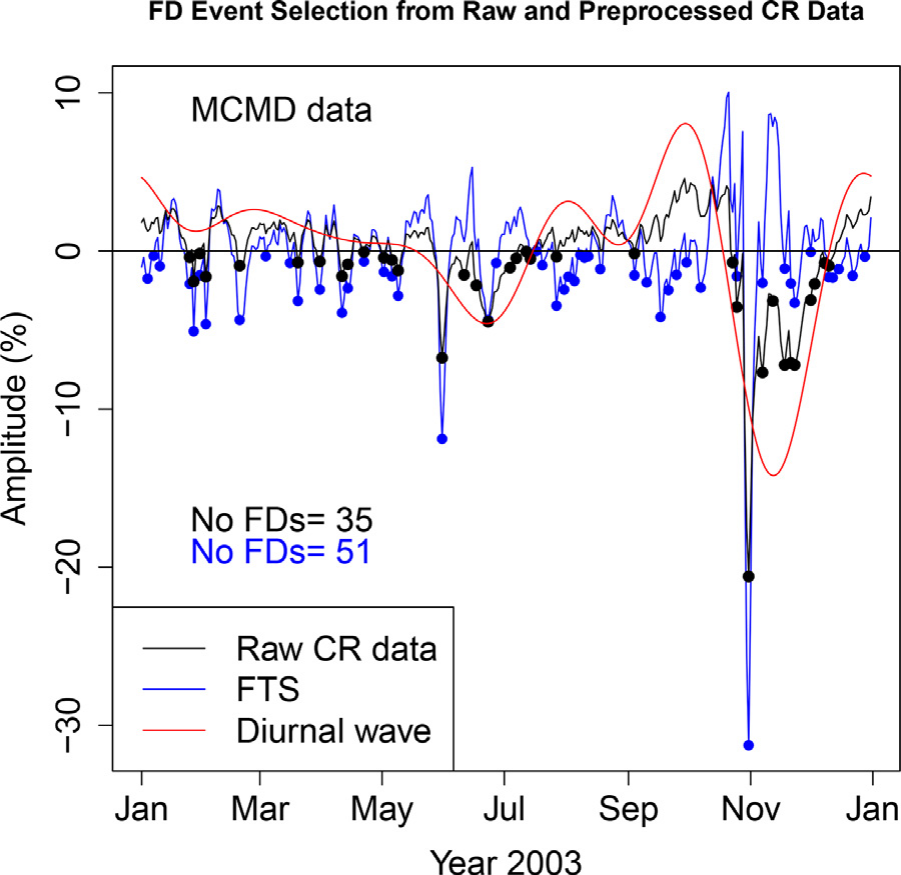
Comparison FDs selected from raw and preliminary processed MCMD CR data. The filled circles are indications of FDs.

**Fig. 4:**
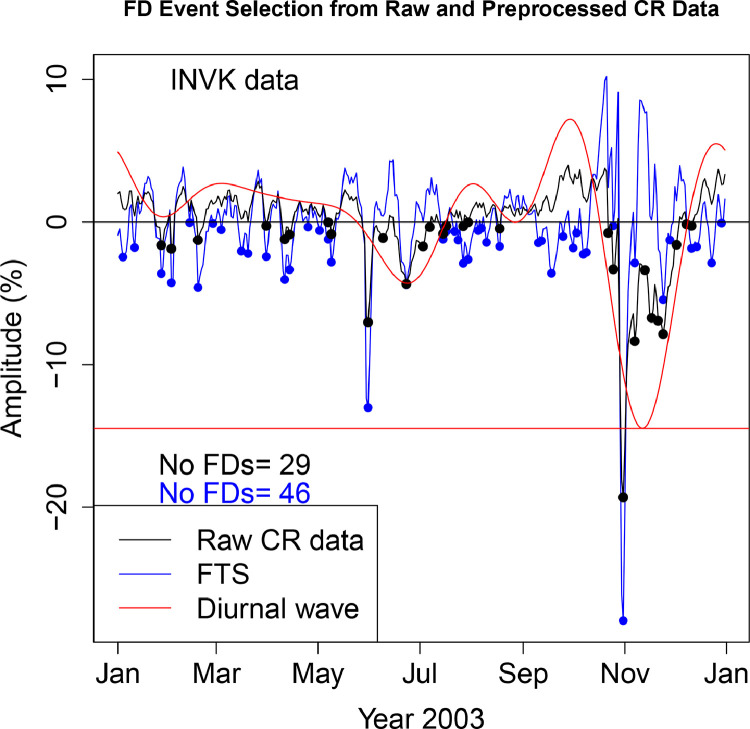
Comparison FDs selected from raw and preliminary processed INVK CR data. The filled circles are indications of FDs.

**Fig. 5:**
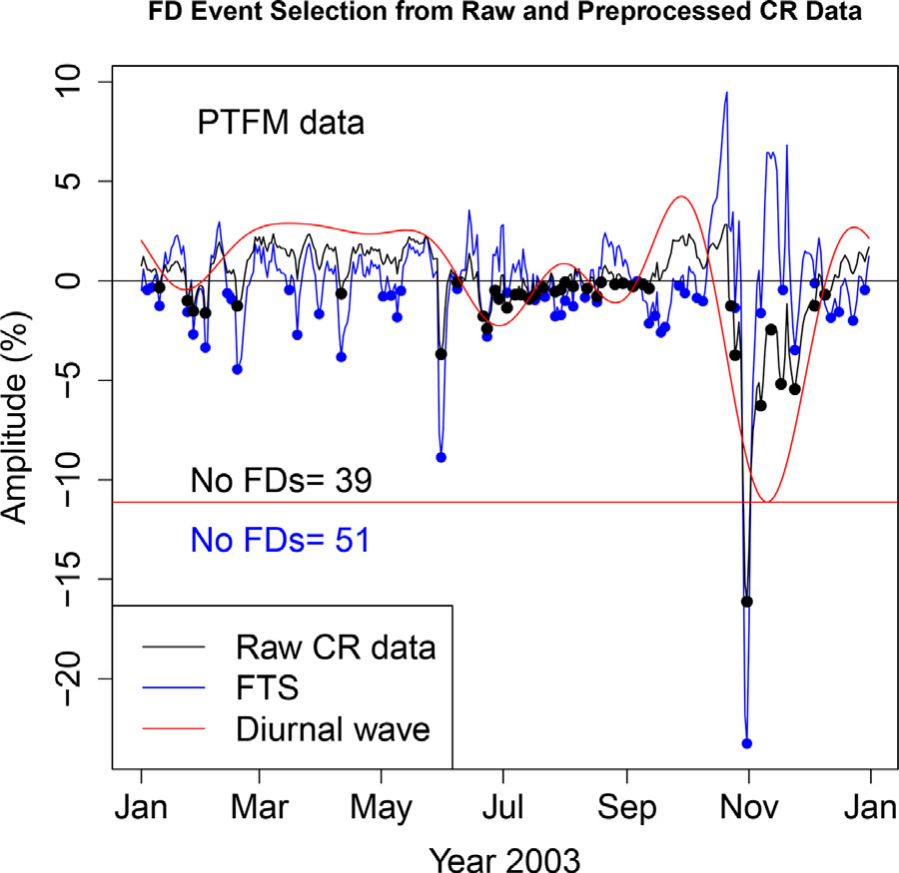
Comparison FDs selected from raw and preliminary processed MGDN CR data. The filled circles are indications of FDs.

**Fig. 6:**
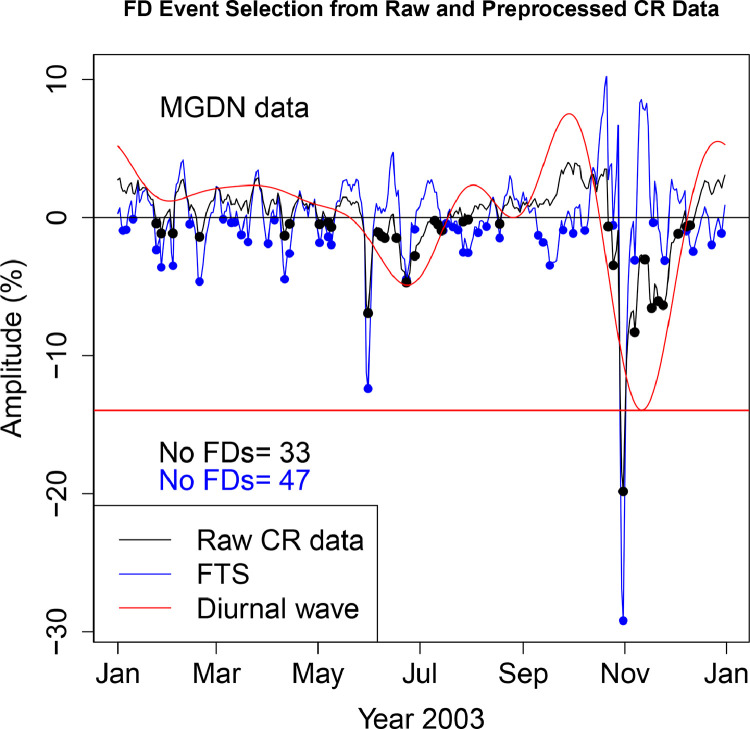
Comparison FDs selected from raw and preliminary processed MGDN CR data. The filled circles are indications of FDs.

**Fig. 7:**
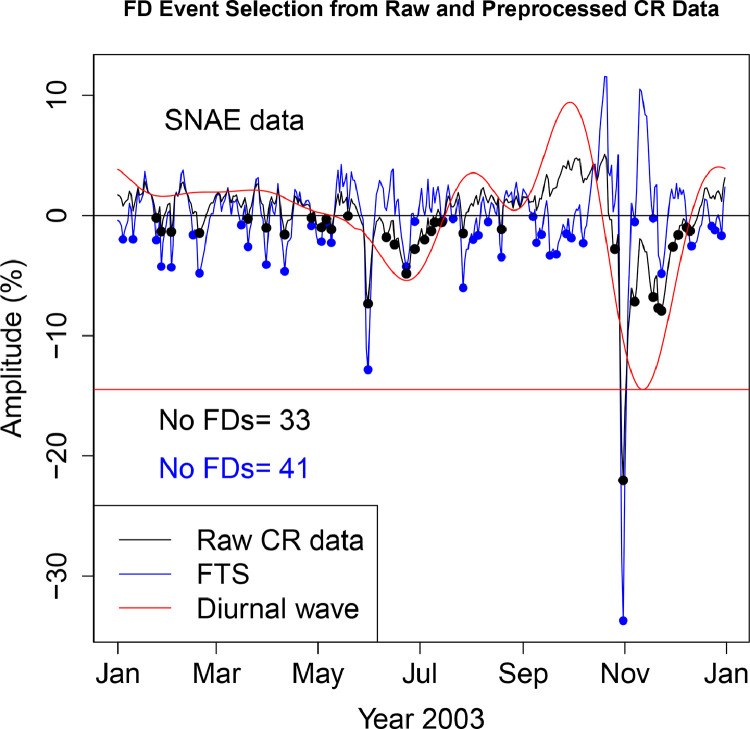
Comparison FDs selected from raw and preliminary processed SNAE CR data. The filled circles are indications of FDs.

**Fig. 8:**
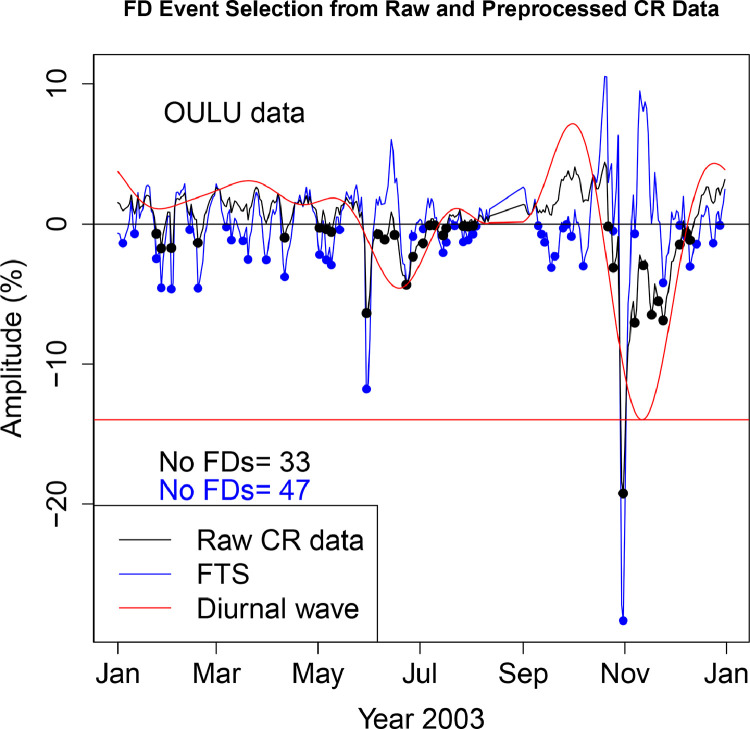
Comparison FDs selected from raw and preliminary processed OULU CR data. The filled circles are indications of FDs.

**Fig. 9:**
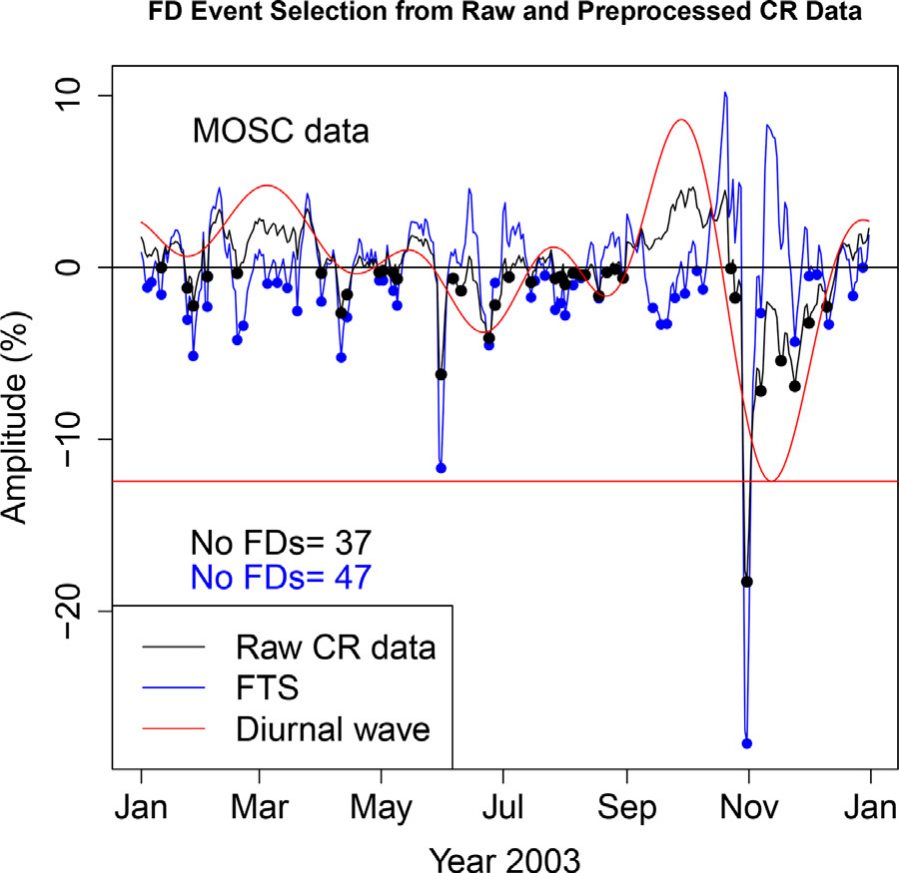
Comparison FDs selected from raw and preliminary processed MOSC CR data. The filled circles are indications of FDs.

The FD data presented in Table 1, Table 2, Table 3, Table 4, Table 5, Table 6 are selected from Fourier transformed CR data. The Fourier transformed data are labeled ”FTS” in the Figures. The FTS data are represented by blue lines and filled circles. The blue filled circles represent the FDs associated with the FTS. The number of FDs detected from FTS is displayed in the blue filled circles in Fig. 1, Fig. 2, Fig. 3, Fig. 4, Fig. 5, Fig. 6, Fig. 7, Fig. 8, Fig. 9. The method of processing the raw CR data and the attempted automated FD event identification are explained in the ”R-Fourier-FD Location Algorithm” section and in Fig. 11.

## 3 Catalogues of Forbush Decreases Identified from Unprocessed CR Data

**Fig. 10:**
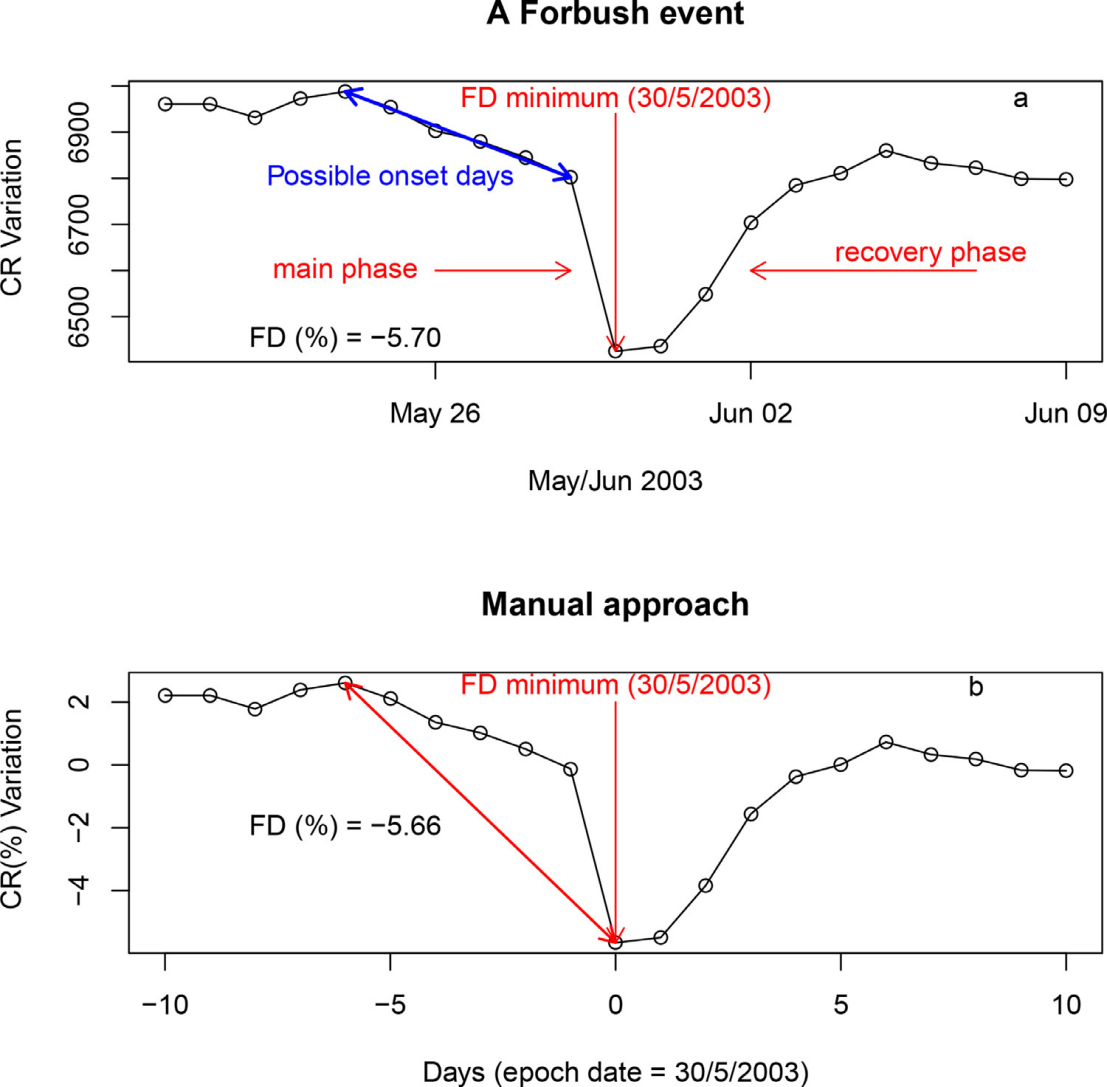
Illustration of the manual method of FD event identification (APTY station).

These are presented in Tables 7 and 8. The unprocessed data here are CR data where the influence of CR diurnal anisotropy has not been removed. Although some data transformation or preprocessing are also implemented here, as indicated in Fig. 10, the CR data are referred to as unprocessed since the bias arising from CR anisotropy has not been corrected. Magnitudes of events here are comparable to those identified by other researchers employing manual identification. The advantage lies in the much greater number of events than those identified manually in 2003.

**Table 7:** Catalogues of Forbush Decreases Identified from Unprocessed CR Data at APTY, MCMD, MOSC, OULU and SNAE CR stations.

	Date	APTY	Date	MCMD	Date	MOSC	Date	OULU	Date	SNAE
1	2003-01-24	−1.18	2003-01-25	−0.38	2003-01-11	−0.00	2003-01-24	−0.70	2003-01-24	−0.20
2	2003-01-27	−2.18	2003-01-27	−1.91	2003-01-24	−1.19	2003-01-27	−1.75	2003-01-27	−1.32
3	2003-02-03	−1.96	2003-01-30	−0.13	2003-01-27	−2.22	2003-02-02	−1.71	2003-02-02	−1.34
4	2003-02-18	−1.33	2003-02-02	−1.62	2003-02-03	−0.51	2003-02-18	−1.34	2003-02-19	−1.43
5	2003-03-20	−1.68	2003-02-19	−0.94	2003-02-18	−0.33	2003-04-11	−0.96	2003-03-20	−0.24
6	2003-03-31	−1.62	2003-03-20	−0.72	2003-04-01	−0.34	2003-05-02	−0.28	2003-03-31	−1.02
7	2003-04-05	−0.23	2003-03-31	−0.65	2003-04-11	−2.64	2003-05-06	−0.38	2003-04-11	−1.58
8	2003-04-10	−2.38	2003-04-11	−1.57	2003-04-14	−1.57	2003-05-09	−0.54	2003-04-27	−0.20
9	2003-05-02	−0.33	2003-04-14	−0.84	2003-04-30	−0.28	2003-05-30	−6.37	2003-05-03	−0.98
10	2003-05-06	−0.47	2003-04-22	−0.06	2003-05-02	−0.20	2003-06-06	−0.73	2003-05-06	−0.28
11	2003-05-09	−0.26	2003-05-02	−0.42	2003-05-07	−0.31	2003-06-10	−1.12	2003-05-09	−1.13
12	2003-05-30	−6.09	2003-05-06	−0.57	2003-05-09	−0.67	2003-06-16	−0.78	2003-05-19	−0.01
13	2003-06-10	−0.89	2003-05-09	−1.22	2003-05-31	−6.21	2003-06-23	−4.32	2003-05-31	−7.34
14	2003-06-16	−0.41	2003-05-31	−6.75	2003-06-06	−0.65	2003-06-27	−2.33	2003-06-11	−1.79
15	2003-06-23	−3.90	2003-06-11	−1.48	2003-06-10	−1.34	2003-07-03	−1.36	2003-06-16	−2.40
16	2003-06-27	−1.96	2003-06-17	−2.15	2003-06-24	−4.10	2003-07-07	−0.08	2003-06-23	−4.82
17	2003-07-03	−1.05	2003-06-23	−4.47	2003-06-27	−2.17	2003-07-10	−0.08	2003-06-28	−2.79
18	2003-07-14	−0.49	2003-07-04	−1.05	2003-07-04	−0.56	2003-07-15	−0.80	2003-07-04	−1.98
19	2003-07-17	−0.30	2003-07-07	−0.45	2003-07-15	−0.85	2003-07-17	−0.29	2003-07-08	−1.26
20	2003-07-27	−0.04	2003-07-12	−0.04	2003-07-27	−0.63	2003-07-27	−0.15	2003-07-10	−0.54
21	2003-07-30	−0.10	2003-07-14	−0.47	2003-07-30	−0.51	2003-07-30	−0.19	2003-07-14	−0.54
22	2003-08-18	−0.32	2003-07-27	−0.35	2003-08-01	−0.95	2003-08-02	−0.12	2003-07-27	−1.47
23	2003-10-22	−0.57	2003-09-04	−0.14	2003-08-05	−0.34	2003-10-22	−0.19	2003-08-19	−1.15
24	2003-10-25	−3.30	2003-10-23	−0.72	2003-08-09	−0.45	2003-10-25	−3.13	2003-10-26	−2.79
25	2003-10-31	−19.76	2003-10-25	−3.55	2003-08-11	−0.45	2003-10-31	−19.25	2003-10-31	−22.04
26	2003-11-07	−7.73	2003-10-31	−20.59	2003-08-18	−1.66	2003-11-07	−7.06	2003-11-07	−7.15
27	2003-11-12	−2.80	2003-11-07	−7.68	2003-08-22	−0.27	2003-11-12	−2.96	2003-11-18	−6.77
28	2003-11-17	−7.04	2003-11-12	−3.16	2003-08-26	−0.10	2003-11-17	−6.48	2003-11-21	−7.70
29	2003-11-21	−6.09	2003-11-18	−7.21	2003-08-30	−0.61	2003-11-21	−5.51	2003-11-23	−7.92
30	2003-11-24	−7.26	2003-11-21	−7.08	2003-10-23	−0.05	2003-11-24	−6.88	2003-11-30	−2.59
31	2003-12-04	−1.91	2003-11-23	−7.21	2003-10-25	−1.77	2003-12-04	−1.45	2003-12-03	−1.60
32	2003-12-08	−1.39	2003-12-01	−3.08	2003-10-31	−18.26	2003-12-08	−0.63	2003-12-08	−0.98
33	2003-12-11	−2.26	2003-12-03	−2.06	2003-11-07	−7.20	2003-12-10	−1.15	2003-12-10	−1.26
34	2003-12-14	−0.44	2003-12-08	−0.74	2003-11-17	−5.43				
35			2003-12-10	−0.92	2003-11-24	−6.92				
36					2003-12-01	−3.22				
37					2003-12-10	−2.28				
38	365 days	−	365 days	−	365 days	−	346 days	−	361 days	−

**Table 8:** Catalogues of Forbush Decreases Identified from Unprocessed CR Data at INVK, NGDN, PTFM and SOPO stations. Part of the data are reproduced from table 4 of [6].

	Date	INVK	Date	MGDN	Date	PTFM	Date	SOPO
1	2003-01-27	−1.62	2003-01-24	−0.42	2003-01-10	−0.32	2003-01-27	−2.03
2	2003-02-02	−1.86	2003-01-27	−1.14	2003-01-24	−1.00	2003-02-02	−1.08
3	2003-02-18	−1.26	2003-02-03	−1.13	2003-01-27	−1.51	2003-02-18	−0.92
4	2003-03-31	−0.25	2003-02-19	−1.40	2003-02-02	−1.61	2003-02-20	−0.91
5	2003-04-11	−1.19	2003-04-11	−1.30	2003-02-18	−1.25	2003-03-20	−0.55
6	2003-04-14	−0.87	2003-04-14	−0.45	2003-04-11	−0.63	2003-03-31	−0.28
7	2003-05-07	−0.01	2003-05-02	−0.47	2003-05-31	−3.68	2003-04-11	−1.78
8	2003-05-09	−0.84	2003-05-07	−0.34	2003-06-08	−0.07	2003-04-14	−0.76
9	2003-05-31	−7.01	2003-05-09	−0.67	2003-06-21	−1.77	2003-05-02	−0.42
10	2003-06-09	−1.13	2003-05-31	−6.93	2003-06-23	−2.39	2003-05-06	−0.35
11	2003-06-23	−4.35	2003-06-06	−1.04	2003-06-27	−0.48	2003-05-09	−1.13
12	2003-07-03	−1.71	2003-06-08	−1.34	2003-06-29	−0.89	2003-05-31	−8.68
13	2003-07-07	−0.33	2003-06-10	−1.46	2003-07-03	−1.36	2003-06-06	−0.76
14	2003-07-15	−0.80	2003-06-17	−1.48	2003-07-07	−0.69	2003-06-11	−1.61
15	2003-07-17	−0.25	2003-06-23	−4.67	2003-07-10	−0.69	2003-06-17	−2.00
16	2003-07-27	−0.30	2003-06-28	−2.77	2003-07-14	−0.89	2003-06-23	−5.92
17	2003-07-30	−0.02	2003-07-10	−0.23	2003-07-17	−0.69	2003-07-04	−1.30
18	2003-08-18	−0.45	2003-07-12	−0.51	2003-07-21	−0.32	2003-07-07	−0.72
19	2003-10-22	−0.77	2003-07-14	−0.92	2003-07-27	−0.53	2003-07-14	−0.78
20	2003-10-25	−3.31	2003-07-27	−0.28	2003-07-30	−0.43	2003-07-27	−0.43
21	2003-10-31	−19.29	2003-07-30	−0.14	2003-08-01	−0.07	2003-08-18	−0.59
22	2003-11-07	−8.35	2003-08-18	−0.43	2003-08-05	−0.27	2003-10-23	−0.99
23	2003-11-13	−3.37	2003-10-22	−0.64	2003-08-12	−0.38	2003-10-25	−3.70
24	2003-11-17	−6.72	2003-10-25	−3.46	2003-08-17	−0.79	2003-10-31	−25.07
25	2003-11-21	−6.92	2003-10-31	−19.83	2003-08-19	−0.07	2003-11-07	−9.30
26	2003-11-24	−7.84	2003-11-07	−8.28	2003-08-26	−0.17	2003-11-18	−9.17
27	2003-12-02	−1.58	2003-11-13	−3.04	2003-08-30	−0.12	2003-11-21	−9.71
28	2003-12-08	−0.15	2003-11-17	−6.54	2003-09-04	−0.27	2003-11-23	−9.64
29	2003-12-11	−0.25	2003-11-21	−6.05	2003-09-09	−0.17	2003-12-03	−2.76
30			2003-11-24	−6.35	2003-09-12	−0.38	2003-12-08	−1.12
31			2003-12-03	−1.15	2003-10-23	−1.25	2003-12-10	−1.55
32			2003-12-08	−0.65	2003-10-25	−3.73		
33			2003-12-10	−0.56	2003-10-31	−16.12		
34					2003-11-07	−6.26		
35					2003-11-12	−2.44		
36					2003-11-17	−5.18		
37					2003-11-24	−5.43		
38					2003-12-04	−1.25		
39					2003-12-09	−0.69		
40	365 days	−	365 days	−	365 days	−	365 days	−

**Fig. 11:**
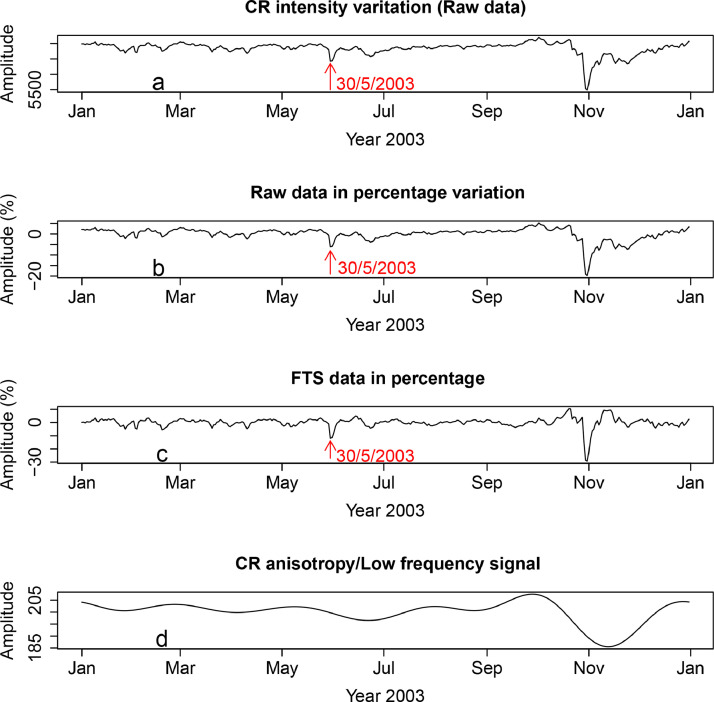
Illustration of the automated method of FD event identification (APTY station).

Additionally the magnitude of those FDs identified from the unprocessed data is on average less than those identified using the FTS method.

## 4 Catalogues of Forbush Decreases Identified from FTS (FD1), Unprocessed (FD2) CR Data and the Amplitude of The Associated Anisotropy

Although FDs and concurrent anisotropies have been well investigated, the magnitudes of anisotropies in individual NMs have not been quantified. The IZMIRAN group attempted this using data from an array of NMs (see http://spaceweather.izmiran.ru/eng/fds1965.html). Adjustment for the influence of anisotropies on the magnitude of the FDs has not been included. In the recent work of [6], two sets of FDs are calculated; FD1 (where the contribution from anisotropy is removed), and FD2 (where the impact of anisotropy is not taken into account). The amplitudes of both the normal and the enhanced anisotropies accompanying each FD at the ten stations are presented in Tables 9–18. The two FD datasets (FD1 and FD2) represetned by the black and blue filled circles in Fig. 1, Fig. 2, Fig. 3, Fig. 4, Fig. 5, Fig. 6, Fig. 7, Fig. 8, Fig. 9 are also presented. Relating the absolute magnitudes of the anisotropies with the magnitude of two FD datasets at the different stations could lead to the understanding of the dependence of CR anisotropies on terrestrial/local factors.

**Table 9:** Catalogues of Forbush Decreases Identified from FTS and Unprocessed CR Data at CLMX station and the associated amplitude of the CR anisotropy (ADV). Part of the data are reproduced from table 4 of [6].

Order	Date1	FD1 (%)	ADV (%)	Date2	FD2 (%)	ADV (%)
1	2003-01-04	−2.46	4.52	2003-01-24	−0.36	1.42
2	2003-01-10	−1.77	3.18	2003-01-27	−2.23	1.45
3	2003-01-24	−2.14	1.42	2003-02-02	−1.09	1.85
4	2003-01-27	−5.92	1.45	2003-02-18	−1.45	3.43
5	2003-02-02	−4.03	1.85	2003-03-20	−0.47	2.72
6	2003-02-13	−0.52	3.02	2003-03-31	−0.28	1.93
7	2003-02-18	−6.32	3.43	2003-04-11	−2.10	1.06
8	2003-03-10	−0.62	3.34	2003-04-24	−0.68	0.21
9	2003-03-12	−0.24	3.22	2003-04-29	−0.14	0.04
10	2003-03-16	−1.35	2.98	2003-05-02	−0.87	−0.02
11	2003-03-20	−3.65	2.72	2003-05-06	−0.47	−0.06
12	2003-03-31	−2.48	1.93	2003-05-09	−1.09	−0.09
13	2003-04-11	−5.26	1.06	2003-05-31	−8.30	−1.98
14	2003-04-24	−1.57	0.21	2003-06-06	−1.12	−3.24
15	2003-04-29	−0.32	0.04	2003-06-11	−1.88	−4.35
16	2003-05-02	−1.73	−0.02	2003-06-16	−1.77	−5.28
17	2003-05-06	−0.87	−0.06	2003-06-23	−5.75	−5.92
18	2003-05-09	−2.10	−0.09	2003-06-30	−2.51	−5.41
19	2003-05-31	−14.63	−1.98	2003-07-03	−1.72	−4.82
20	2003-06-23	−5.57	−5.92	2003-07-10	−0.68	−2.75
21	2003-07-14	−0.91	−1.33	2003-07-14	−1.12	−1.33
22	2003-07-23	−1.00	1.59	2003-07-27	−0.60	2.44
23	2003-07-27	−3.64	2.44	2003-07-30	−0.17	2.80
24	2003-07-30	−3.13	2.80	2003-10-23	−0.74	−5.03
25	2003-08-05	−1.65	2.78	2003-10-25	−2.97	−6.88
26	2003-08-07	−0.47	2.59	2003-10-31	−23.17	−11.80
27	2003-08-09	−1.17	2.31	2003-11-07	−8.28	−15.31
28	2003-08-11	−1.22	1.97	2003-11-12	−3.00	−15.84
29	2003-08-17	−0.23	0.82	2003-11-18	−7.49	−14.26
30	2003-09-05	−0.46	1.33	2003-11-21	−7.00	−12.69
31	2003-09-12	−2.05	4.05	2003-11-24	−7.81	−10.73
32	2003-09-15	−1.66	5.41	2003-11-30	−2.94	−6.11
33	2003-09-18	−3.47	6.73	2003-12-10	−0.19	1.28
34	2003-09-21	−3.17	7.90			
35	2003-09-26	−0.56	9.21			
36	2003-09-30	−1.38	9.43			
37	2003-10-07	−2.25	7.63			
38	2003-10-09	−2.25	6.60			
39	2003-10-31	−34.53	−11.80			
40	2003-11-07	−1.25	−15.31			
41	2003-11-18	−0.72	−14.26			
42	2003-11-21	−1.30	−12.69			
43	2003-11-24	−4.90	−10.73			
44	2003-12-10	−1.67	1.28			
45	2003-12-12	−1.35	2.43			
46	2003-12-14	−1.41	3.42			
47	2003-12-16	−1.15	4.24			
48	2003-12-23	−1.79	5.76

**Table 10:** Catalogues of Forbush Decreases Identified from FTS and Unprocessed CR Data at PTFM station and the associated amplitude of the CR anisotropy (ADV). Part of the data are reproduced from table 4 of [6].

Order	Date1	FD1 (%)	ADV (%)	Date2	FD2 (%)	ADV (%)
1	2003-01-04	−0.45	1.55	2003-01-10	−0.32	0.60
2	2003-01-06	−0.33	1.23	2003-01-24	−1.00	−0.43
3	2003-01-10	−1.25	0.60	2003-01-27	−1.51	−0.34
4	2003-01-24	−1.56	−0.43	2003-02-02	−1.61	0.13
5	2003-01-27	−2.69	−0.34	2003-02-18	−1.25	1.93
6	2003-02-02	−3.36	0.13	2003-04-11	−0.63	2.55
7	2003-02-13	−0.60	1.40	2003-05-31	−3.68	1.51
8	2003-02-15	−0.93	1.62	2003-06-08	−0.07	0.25
9	2003-02-18	−4.43	1.93	2003-06-21	−1.77	−1.79
10	2003-03-16	−0.45	2.89	2003-06-23	−2.39	−1.99
11	2003-03-20	−2.71	2.88	2003-06-27	−0.48	−2.22
12	2003-03-31	−1.67	2.77	2003-06-29	−0.89	−2.25
13	2003-04-11	−3.82	2.55	2003-07-03	−1.36	−2.14
14	2003-05-02	−0.78	2.40	2003-07-07	−0.69	−1.81
15	2003-05-06	−0.74	2.46	2003-07-10	−0.69	−1.45
16	2003-05-09	−1.81	2.51	2003-07-14	−0.89	−0.88
17	2003-05-11	−0.50	2.53	2003-07-17	−0.69	−0.43
18	2003-05-31	−8.87	1.51	2003-07-21	−0.32	0.13
19	2003-06-08	−0.39	0.25	2003-07-27	−0.53	0.72
20	2003-06-21	−1.75	−1.79	2003-07-30	−0.43	0.85
21	2003-06-23	−2.79	−1.99	2003-08-01	−0.07	0.86
22	2003-07-03	−0.58	−2.14	2003-08-05	−0.27	0.72
23	2003-07-14	−0.91	−0.88	2003-08-12	−0.38	0.07
24	2003-07-17	−0.94	−0.43	2003-08-17	−0.79	−0.51
25	2003-07-22	−0.80	0.26	2003-08-19	−0.07	−0.72
26	2003-07-27	−1.78	0.72	2003-08-26	−0.17	−1.10
27	2003-07-30	−1.70	0.85	2003-08-30	−0.12	−0.97
28	2003-08-01	−0.99	0.86	2003-09-04	−0.27	−0.37
29	2003-08-05	−1.27	0.72	2003-09-09	−0.17	0.64
30	2003-08-11	−0.84	0.19	2003-09-12	−0.38	1.37
31	2003-08-17	−1.07	−0.51	2003-10-23	−1.25	−4.99
32	2003-09-04	−0.17	−0.37	2003-10-25	−3.73	−6.10
33	2003-09-06	−0.02	−0.01	2003-10-31	−16.12	−9.00
34	2003-09-12	−2.12	1.37	2003-11-07	−6.26	−10.91
35	2003-09-15	−1.76	2.14	2003-11-12	−2.44	−11.04
36	2003-09-18	−2.60	2.88	2003-11-17	−5.18	−10.11
37	2003-09-20	−2.31	3.32	2003-11-24	−5.43	−7.42
38	2003-09-27	−0.23	4.22	2003-12-04	−1.25	−2.40
39	2003-09-30	−0.61	4.19	2003-12-09	−0.69	−0.18
40	2003-10-06	−0.85	3.20			
41	2003-10-09	−1.02	2.23			
42	2003-10-25	−1.36	−6.10			
43	2003-10-31	−23.24	−9.00			
44	2003-11-07	−1.61	−10.91			
45	2003-11-18	−0.44	−9.81			
46	2003-11-24	−3.45	−7.42			
47	2003-12-04	−0.11	−2.40			
48	2003-12-12	−1.85	0.89			
49	2003-12-16	−1.56	1.94			
50	2003-12-23	−2.00	2.69			
51	2003-12-29	−0.45	2.38

**Table 11:** Catalogues of Forbush Decreases Identified from FTS and Unprocessed CR Data at SOPO station and the associated amplitude of the CR anisotropy (ADV). Part of the data are reproduced from table 4 of [6].

Order	Date1	FD1 (%)	ADV (%)	Date2	FD2 (%)	ADV (%)
1	2003-01-04	−2.64	4.77	2003-01-27	−2.03	2.06
2	2003-01-10	−1.96	3.79	2003-02-02	−1.08	2.31
3	2003-01-25	−2.00	2.07	2003-02-18	−0.92	3.71
4	2003-01-27	−6.12	2.06	2003-02-20	−0.91	3.82
5	2003-02-02	−4.48	2.31	2003-03-20	−0.55	2.89
6	2003-02-20	−5.64	3.82	2003-03-31	−0.28	2.20
7	2003-02-27	−0.08	3.97	2003-04-11	−1.78	1.69
8	2003-03-04	−0.03	3.86	2003-04-14	−0.76	1.58
9	2003-03-10	−0.01	3.56	2003-05-02	−0.42	1.20
10	2003-03-16	−0.56	3.16	2003-05-06	−0.35	1.13
11	2003-03-20	−3.98	2.89	2003-05-09	−1.13	1.05
12	2003-03-31	−2.76	2.20	2003-05-31	−8.68	−1.66
13	2003-04-11	−5.25	1.69	2003-06-06	−0.76	−3.06
14	2003-04-14	−3.10	1.58	2003-06-11	−1.61	−4.21
15	2003-04-22	−0.49	1.36	2003-06-17	−2.00	−5.28
16	2003-05-02	−2.04	1.20	2003-06-23	−5.92	−5.71
17	2003-05-06	−1.84	1.13	2003-07-04	−1.30	−4.25
18	2003-05-09	−3.30	1.05	2003-07-07	−0.72	−3.38
19	2003-05-31	−15.69	−1.66	2003-07-14	−0.78	−0.93
20	2003-06-23	−6.14	−5.71	2003-07-27	−0.43	2.95
21	2003-06-28	−0.37	−5.41	2003-08-18	−0.59	1.42
22	2003-07-14	−0.64	−0.93	2003-10-23	−0.99	−5.08
23	2003-07-20	−0.59	1.17	2003-10-25	−3.70	−7.03
24	2003-07-23	−0.27	2.06	2003-10-31	−25.07	−12.38
25	2003-07-27	−3.81	2.95	2003-11-07	−9.30	−16.62
26	2003-07-30	−2.83	3.35	2003-11-18	−9.17	−16.83
27	2003-08-05	−1.67	3.44	2003-11-21	−9.71	−15.52
28	2003-08-10	−1.07	2.88	2003-11-23	−9.64	−14.39
29	2003-08-16	−0.26	1.79	2003-12-03	−2.76	−6.84
30	2003-08-18	−2.60	1.42	2003-12-08	−1.12	−2.86
31	2003-09-05	−0.06	1.68	2003-12-10	−1.55	−1.39
32	2003-09-12	−1.96	4.13			
33	2003-09-17	−5.04	6.22			
34	2003-09-21	−3.40	7.74			
35	2003-09-26	−1.18	9.03			
36	2003-09-30	−1.85	9.29			
37	2003-10-07	−2.98	7.62			
38	2003-10-25	−0.37	−7.03			
39	2003-10-31	−37.77	−12.38			
40	2003-11-07	−1.98	−16.62			
41	2003-11-18	−1.52	−16.83			
42	2003-11-21	−3.90	−15.52			
43	2003-11-24	−4.95	−13.75			
44	2003-12-10	−1.70	−1.39			
45	2003-12-12	−1.74	−0.04			
46	2003-12-15	−1.40	1.73			
47	2003-12-23	−1.45	4.68			
48	2003-12-28	−0.80	5.23

**Table 12:** Catalogues of Forbush Decreases Identified from FTS and Unprocessed CR Data at APTY station and the associated amplitude of the CR anisotropy (ADV).

Order	Date1	FD1 (%)	ADV (%)	Date2	FD2 (%)	ADV (%)
1	2003-01-03	−0.28	3.88	2003-01-24	−1.18	0.63
2	2003-01-24	−3.00	0.63	2003-01-27	−2.18	0.59
3	2003-01-27	−4.94	0.59	2003-02-03	−1.96	1.02
4	2003-02-03	−4.93	1.02	2003-02-18	−1.33	2.86
5	2003-02-18	−5.52	2.86	2003-03-20	−1.68	0.97
6	2003-03-20	−4.33	0.97	2003-03-31	−1.62	−0.06
7	2003-03-31	−3.18	−0.06	2003-04-05	−0.23	−0.15
8	2003-04-05	−0.31	−0.15	2003-04-10	−2.38	0.02
9	2003-04-10	−4.78	0.02	2003-05-02	−0.33	2.04
10	2003-05-02	−2.71	2.04	2003-05-06	−0.47	2.22
11	2003-05-06	−3.15	2.22	2003-05-09	−0.26	2.25
12	2003-05-09	−2.78	2.25	2003-05-30	−6.09	−0.26
13	2003-05-14	−0.35	2.08	2003-06-10	−0.89	−2.43
14	2003-05-30	−11.92	−0.26	2003-06-16	−0.41	−3.22
15	2003-06-23	−4.32	−3.48	2003-06-23	−3.90	−3.48
16	2003-06-27	−0.66	−3.25	2003-06-27	−1.96	−3.25
17	2003-07-15	−0.98	0.20	2003-07-03	−1.05	−2.43
18	2003-07-17	−1.24	0.63	2003-07-14	−0.49	−0.03
19	2003-07-24	−0.53	1.85	2003-07-17	−0.30	0.63
20	2003-07-27	−2.24	2.16	2003-07-27	−0.04	2.16
21	2003-07-30	−2.52	2.32	2003-07-30	−0.10	2.32
22	2003-08-05	−0.25	2.21	2003-08-18	−0.32	0.93
23	2003-08-10	−0.14	1.78	2003-10-22	−0.57	−3.71
24	2003-08-18	−1.57	0.93	2003-10-25	−3.30	−6.03
25	2003-09-06	−0.09	2.29	2003-10-31	−19.76	−10.26
26	2003-09-10	−1.51	3.41	2003-11-07	−7.73	−13.59
27	2003-09-12	−1.71	4.03	2003-11-12	−2.80	−14.46
28	2003-09-14	−2.34	4.66	2003-11-17	−7.04	−13.95
29	2003-09-18	−3.81	5.86	2003-11-21	−6.09	−12.62
30	2003-09-22	−2.63	6.84	2003-11-24	−7.26	−11.17
31	2003-09-30	−0.34	7.42	2003-12-04	−1.91	−4.78
32	2003-10-08	−1.87	5.41	2003-12-08	−1.39	−2.14
33	2003-10-25	−0.57	−6.03	2003-12-11	−2.26	−0.34
34	2003-10-31	−29.26	−10.26	2003-12-14	−0.44	1.22
35	2003-11-07	−1.87	−13.59			
36	2003-11-17	−0.13	−13.95			
37	2003-11-24	−3.36	−11.17			
38	2003-12-08	−0.63	−2.14			
39	2003-12-11	−4.18	−0.34			
40	2003-12-15	−2.40	1.68			
41	2003-12-19	−2.16	3.16			
42	2003-12-23	−2.33	4.06			
43	2003-12-25	−3.53	4.30			
44	2003-12-28	−2.26	4.40

**Table 13:** Catalogues of Forbush Decreases Identified from FTS and Unprocessed CR Data at INVK station and the associated amplitude of the CR anisotropy (ADV). Part of the data are reproduced from table 4 of [6].

Order	Date1	FD1 (%)	ADV (%)	Date2	FD2 (%)	ADV (%)
1	2003-01-04	−2.45	4.32	2003-01-27	−1.62	0.38
2	2003-01-11	−1.80	2.66	2003-02-02	−1.86	0.52
3	2003-01-27	−3.61	0.38	2003-02-18	−1.26	2.07
4	2003-02-02	−4.24	0.52	2003-03-31	−0.25	1.93
5	2003-02-13	−0.03	1.57	2003-04-11	−1.19	1.65
6	2003-02-18	−4.59	2.07	2003-04-14	−0.87	1.59
7	2003-02-27	−0.10	2.62	2003-05-07	−0.01	1.19
8	2003-03-04	−0.54	2.70	2003-05-09	−0.84	1.13
9	2003-03-16	−2.03	2.44	2003-05-31	−7.01	−1.02
10	2003-03-20	−2.21	2.30	2003-06-09	−1.13	−2.68
11	2003-03-31	−2.44	1.93	2003-06-23	−4.35	−4.29
12	2003-04-11	−4.04	1.65	2003-07-03	−1.71	−3.43
13	2003-04-14	−3.33	1.59	2003-07-07	−0.33	−2.58
14	2003-04-25	−0.33	1.39	2003-07-15	−0.80	−0.42
15	2003-05-02	−0.59	1.29	2003-07-17	−0.25	0.14
16	2003-05-07	−1.20	1.19	2003-07-27	−0.30	2.29
17	2003-05-09	−2.80	1.13	2003-07-30	−0.02	2.58
18	2003-05-31	−13.01	−1.02	2003-08-18	−0.45	0.82
19	2003-06-23	−4.40	−4.29	2003-10-22	−0.77	−3.97
20	2003-07-15	−1.19	−0.42	2003-10-25	−3.31	−6.36
21	2003-07-17	−0.64	0.14	2003-10-31	−19.29	−10.64
22	2003-07-22	−0.66	1.40	2003-11-07	−8.35	−13.84
23	2003-07-24	−1.27	1.81	2003-11-13	−3.37	−14.40
24	2003-07-27	−2.88	2.29	2003-11-17	−6.72	−13.59
25	2003-07-30	−2.63	2.58	2003-11-21	−6.92	−11.93
26	2003-08-05	−0.59	2.59	2003-11-24	−7.84	−10.23
27	2003-08-07	−0.43	2.43	2003-12-02	−1.58	−4.67
28	2003-08-10	−1.41	2.08	2003-12-08	−0.15	−0.47
29	2003-08-18	−1.71	0.82	2003-12-11	−0.25	1.34
30	2003-09-10	−1.45	2.31			
31	2003-09-12	−1.32	2.97			
32	2003-09-18	−3.57	5.02			
33	2003-09-25	−1.00	6.84			
34	2003-10-01	−1.80	7.12			
35	2003-10-03	−0.76	6.86			
36	2003-10-07	−2.26	5.76			
37	2003-10-09	−2.11	4.92			
38	2003-10-25	−0.26	−6.36			
39	2003-10-31	−27.94	−10.64			
40	2003-11-07	−2.85	−13.84			
41	2003-11-24	−5.45	−10.23			
42	2003-11-28	−1.26	−7.56			
43	2003-12-11	−1.84	1.34			
44	2003-12-14	−1.74	2.87			
45	2003-12-23	−2.86	5.35			
46	2003-12-29	−0.06	5.31

**Table 14:** Catalogues of Forbush Decreases Identified from FTS and Unprocessed CR Data at MCMD station and the associated amplitude of the CR anisotropy (ADV).

Order	Date1	FD1 (%)	ADV (%)	Date2	FD2 (%)	ADV (%)
1	2003-01-04	−1.74	4.25	2003-01-25	−0.38	1.31
2	2003-01-07	−0.29	3.75	2003-01-27	−1.91	1.25
3	2003-01-10	−0.97	3.20	2003-01-30	−0.13	1.27
4	2003-01-25	−2.08	1.31	2003-02-02	−1.62	1.37
5	2003-01-27	−5.07	1.25	2003-02-19	−0.94	2.47
6	2003-01-30	−1.53	1.27	2003-03-20	−0.72	1.70
7	2003-02-02	−4.61	1.37	2003-03-31	−0.65	1.14
8	2003-02-19	−4.35	2.47	2003-04-11	−1.57	0.75
9	2003-03-04	−0.32	2.53	2003-04-14	−0.84	0.67
10	2003-03-16	−0.76	1.93	2003-04-22	−0.06	0.54
11	2003-03-20	−3.13	1.70	2003-05-02	−0.42	0.47
12	2003-03-31	−2.44	1.14	2003-05-06	−0.57	0.44
13	2003-04-11	−3.90	0.75	2003-05-09	−1.22	0.40
14	2003-04-14	−2.34	0.67	2003-05-31	−6.75	−1.62
15	2003-04-22	−0.66	0.54	2003-06-11	−1.48	−3.56
16	2003-05-02	−1.31	0.47	2003-06-17	−2.15	−4.34
17	2003-05-06	−1.58	0.44	2003-06-23	−4.47	−4.59
18	2003-05-09	−2.83	0.40	2003-07-04	−1.05	−3.22
19	2003-05-31	−11.87	−1.62	2003-07-07	−0.45	−2.47
20	2003-06-23	−4.36	−4.59	2003-07-12	−0.04	−1.01
21	2003-06-27	−0.74	−4.37	2003-07-14	−0.47	−0.40
22	2003-07-14	−0.53	−0.40	2003-07-27	−0.35	2.75
23	2003-07-17	−0.00	0.50	2003-09-04	−0.14	1.24
24	2003-07-20	−0.90	1.34	2003-10-23	−0.72	−3.90
25	2003-07-27	−3.45	2.75	2003-10-25	−3.55	−5.52
26	2003-07-31	−2.42	3.10	2003-10-31	−20.59	−9.94
27	2003-08-02	−1.60	3.14	2003-11-07	−7.68	−13.37
28	2003-08-05	−1.89	3.04	2003-11-12	−3.16	−14.21
29	2003-08-08	−0.23	2.76	2003-11-18	−7.21	−13.30
30	2003-08-10	−0.41	2.50	2003-11-21	−7.08	−12.14
31	2003-08-12	−0.33	2.20	2003-11-23	−7.21	−11.16
32	2003-08-18	−1.12	1.21	2003-12-01	−3.08	−6.10
33	2003-09-04	−1.53	1.24	2003-12-03	−2.06	−4.72
34	2003-09-10	−1.97	2.91	2003-12-08	−0.74	−1.39
35	2003-09-17	−4.16	5.40	2003-12-10	−0.92	−0.17
36	2003-09-21	−2.44	6.71			
37	2003-09-25	−1.51	7.67			
38	2003-09-30	−0.70	8.06			
39	2003-10-07	−2.31	6.69			
40	2003-10-25	−1.58	−5.52			
41	2003-10-31	−31.25	−9.94			
42	2003-11-07	−2.00	−13.37			
43	2003-11-18	−1.12	−13.30			
44	2003-11-21	−2.02	−12.14			
45	2003-11-23	−3.26	−11.16			
46	2003-12-01	−0.05	−6.10			
47	2003-12-10	−1.66	−0.17			
48	2003-12-12	−1.68	0.93			
49	2003-12-15	−1.15	2.36			
50	2003-12-22	−1.54	4.47			
51	2003-12-28	−0.35	4.91

**Table 15:** Catalogues of Forbush Decreases Identified from FTS and Unprocessed CR Data at MGDN station and the associated amplitude of the CR anisotropy (ADV). Part of the data are reproduced from table 4 of [6].

Order	Date1	FD1 (%)	ADV (%)	Date2	FD2 (%)	ADV (%)
1	2003-01-04	−0.93	4.73	2003-01-24	−0.42	1.50
2	2003-01-06	−0.89	4.38	2003-01-27	−1.14	1.32
3	2003-01-10	−0.09	3.61	2003-02-03	−1.13	1.22
4	2003-01-24	−2.35	1.50	2003-02-19	−1.40	1.81
5	2003-01-27	−3.60	1.32	2003-04-11	−1.30	1.86
6	2003-02-03	−3.48	1.22	2003-04-14	−0.45	1.71
7	2003-02-13	−0.47	1.56	2003-05-02	−0.47	0.87
8	2003-02-19	−4.61	1.81	2003-05-07	−0.34	0.71
9	2003-03-05	−0.10	2.17	2003-05-09	−0.67	0.64
10	2003-03-10	−0.37	2.24	2003-05-31	−6.93	−1.48
11	2003-03-12	−0.34	2.26	2003-06-06	−1.04	−2.61
12	2003-03-16	−1.23	2.30	2003-06-08	−1.34	−3.00
13	2003-03-20	−1.75	2.33	2003-06-10	−1.46	−3.38
14	2003-04-01	−1.87	2.24	2003-06-17	−1.48	−4.49
15	2003-04-05	−0.15	2.12	2003-06-23	−4.67	−4.89
16	2003-04-11	−4.46	1.86	2003-06-28	−2.77	−4.70
17	2003-04-14	−2.60	1.71	2003-07-10	−0.23	−2.29
18	2003-05-02	−1.82	0.87	2003-07-12	−0.51	−1.71
19	2003-05-07	−1.40	0.71	2003-07-14	−0.92	−1.13
20	2003-05-09	−1.97	0.64	2003-07-27	−0.28	1.95
21	2003-05-31	−12.37	−1.48	2003-07-30	−0.14	2.25
22	2003-06-23	−4.45	−4.89	2003-08-18	−0.43	0.62
23	2003-06-28	−0.83	−4.70	2003-10-22	−0.64	−4.03
24	2003-07-15	−0.94	−0.83	2003-10-25	−3.46	−6.37
25	2003-07-17	−0.41	−0.25	2003-10-31	−19.83	−10.50
26	2003-07-21	−0.67	0.81	2003-11-07	−8.28	−13.46
27	2003-07-24	−0.91	1.46	2003-11-13	−3.04	−13.86
28	2003-07-27	−2.51	1.95	2003-11-17	−6.54	−12.99
29	2003-07-30	−2.53	2.25	2003-11-21	−6.05	−11.33
30	2003-08-05	−1.08	2.27	2003-11-24	−6.35	−9.67
31	2003-08-10	−0.64	1.78	2003-12-03	−1.15	−3.63
32	2003-08-18	−1.49	0.62	2003-12-08	−0.65	−0.34
33	2003-09-10	−1.28	2.73	2003-12-10	−0.56	0.84
34	2003-09-13	−1.80	3.77			
35	2003-09-17	−3.45	5.18			
36	2003-09-25	−0.91	7.26			
37	2003-10-01	−1.12	7.39			
38	2003-10-08	−0.92	5.44			
39	2003-10-25	−0.55	−6.37			
40	2003-10-31	−29.17	−10.50			
41	2003-11-07	−3.09	−13.46			
42	2003-11-18	−0.35	−12.65			
43	2003-11-25	−3.12	−9.06			
44	2003-12-08	−0.97	−0.34			
45	2003-12-12	−2.46	1.90			
46	2003-12-23	−1.98	5.31			
47	2003-12-29	−1.13	5.44

**Table 16:** Catalogues of Forbush Decreases Identified from FTS and Unprocessed CR Data at MOSC station and the associated amplitude of the CR anisotropy (ADV).

Order	Date1	FD1 (%)	ADV (%)	Date2	FD2 (%)	ADV (%)
1	2003-01-04	−1.16	2.37	2003-01-11	−0.00	1.55
2	2003-01-06	−0.84	2.16	2003-01-24	−1.19	0.64
3	2003-01-11	−1.56	1.55	2003-01-27	−2.22	0.70
4	2003-01-24	−3.03	0.64	2003-02-03	−0.51	1.27
5	2003-01-27	−5.13	0.70	2003-02-18	−0.33	3.54
6	2003-02-03	−2.29	1.27	2003-04-01	−0.34	1.29
7	2003-02-18	−4.20	3.54	2003-04-11	−2.64	−0.05
8	2003-02-21	−3.37	3.95	2003-04-14	−1.57	−0.25
9	2003-03-05	−0.93	4.77	2003-04-30	−0.28	0.19
10	2003-03-10	−0.88	4.60	2003-05-02	−0.20	0.36
11	2003-03-15	−1.18	4.13	2003-05-07	−0.31	0.75
12	2003-03-20	−2.52	3.42	2003-05-09	−0.67	0.87
13	2003-04-01	−1.98	1.29	2003-05-31	−6.21	−0.76
14	2003-04-11	−5.24	−0.05	2003-06-06	−0.65	−1.96
15	2003-04-14	−2.89	−0.25	2003-06-10	−1.34	−2.71
16	2003-04-30	−0.76	0.19	2003-06-24	−4.10	−3.70
17	2003-05-02	−0.75	0.36	2003-06-27	−2.17	−3.45
18	2003-05-07	−1.36	0.75	2003-07-04	−0.56	−2.33
19	2003-05-09	−2.21	0.87	2003-07-15	−0.85	0.03
20	2003-05-31	−11.66	−0.76	2003-07-27	−0.63	1.17
21	2003-06-24	−4.51	−3.70	2003-07-30	−0.51	1.04
22	2003-06-27	−0.88	−3.45	2003-08-01	−0.95	0.87
23	2003-07-15	−1.74	0.03	2003-08-05	−0.34	0.35
24	2003-07-17	−0.78	0.39	2003-08-09	−0.45	−0.30
25	2003-07-22	−0.48	1.01	2003-08-11	−0.45	−0.64
26	2003-07-27	−2.44	1.17	2003-08-18	−1.66	−1.54
27	2003-07-30	−2.07	1.04	2003-08-22	−0.27	−1.67
28	2003-08-01	−2.77	0.87	2003-08-26	−0.10	−1.39
29	2003-08-05	−1.03	0.35	2003-08-30	−0.61	−0.66
30	2003-08-09	−0.60	−0.30	2003-10-23	−0.05	−3.51
31	2003-08-18	−1.78	−1.54	2003-10-25	−1.77	−4.97
32	2003-08-30	−0.56	−0.66	2003-10-31	−18.26	−8.85
33	2003-09-14	−2.34	4.97	2003-11-07	−7.20	−11.76
34	2003-09-18	−3.32	6.55	2003-11-17	−5.43	−11.91
35	2003-09-21	−3.29	7.52	2003-11-24	−6.92	−9.51
36	2003-09-25	−1.76	8.38	2003-12-01	−3.22	−5.95
37	2003-09-30	−1.52	8.53	2003-12-10	−2.28	−1.32
38	2003-10-06	−0.20	7.19			
39	2003-10-09	−1.28	5.90			
40	2003-10-31	−27.67	−8.85			
41	2003-11-07	−2.63	−11.76			
42	2003-11-24	−4.32	−9.51			
43	2003-12-01	−0.48	−5.95			
44	2003-12-05	−0.42	−3.79			
45	2003-12-11	−3.29	−0.88			
46	2003-12-23	−1.65	2.47			
47	2003-12-28	−0.01	2.76

**Table 17:** Catalogues of Forbush Decreases Identified from FTS and Unprocessed CR Data at OULU station and the associated amplitude of the CR anisotropy (ADV).

Order	Date1	FD1 (%)	ADV (%)	Date2	FD2 (%)	ADV (%)
1	2003-01-04	−1.37	3.27	2003-01-24	−0.70	1.08
2	2003-01-11	−0.70	2.15	2003-01-27	−1.75	1.07
3	2003-01-24	−2.48	1.08	2003-02-02	−1.71	1.22
4	2003-01-27	−4.57	1.07	2003-02-18	−1.34	1.90
5	2003-02-02	−4.64	1.22	2003-04-11	−0.96	1.84
6	2003-02-13	−0.42	1.69	2003-05-02	−0.28	1.64
7	2003-02-18	−4.59	1.90	2003-05-06	−0.38	1.79
8	2003-03-07	−0.21	2.67	2003-05-09	−0.54	1.85
9	2003-03-10	−1.16	2.81	2003-05-30	−6.37	−0.96
10	2003-03-17	−1.18	3.05	2003-06-06	−0.73	−2.80
11	2003-03-20	−2.51	3.08	2003-06-10	−1.12	−3.69
12	2003-03-31	−2.56	2.71	2003-06-16	−0.78	−4.49
13	2003-04-11	−3.76	1.84	2003-06-23	−4.32	−4.35
14	2003-05-02	−2.19	1.64	2003-06-27	−2.33	−3.76
15	2003-05-06	−2.55	1.79	2003-07-03	−1.36	−2.37
16	2003-05-09	−2.93	1.85	2003-07-07	−0.08	−1.32
17	2003-05-14	−0.42	1.76	2003-07-10	−0.08	−0.57
18	2003-05-30	−11.79	−0.96	2003-07-15	−0.80	0.44
19	2003-06-23	−4.29	−4.35	2003-07-17	−0.29	0.72
20	2003-06-27	−0.90	−3.76	2003-07-27	−0.15	0.98
21	2003-07-03	−0.35	−2.37	2003-07-30	−0.19	0.76
22	2003-07-15	−2.04	0.44	2003-08-02	−0.12	0.49
23	2003-07-17	−1.31	0.72	2003-10-22	−0.19	−3.33
24	2003-07-22	−0.13	1.08	2003-10-25	−3.13	−5.78
25	2003-07-27	−1.28	0.98	2003-10-31	−19.25	−10.18
26	2003-07-30	−1.13	0.76	2003-11-07	−7.06	−13.42
27	2003-08-02	−0.73	0.49	2003-11-12	−2.96	−13.98
28	2003-08-04	−0.17	0.32	2003-11-17	−6.48	−13.01
29	2003-09-10	−0.12	1.74	2003-11-21	−5.51	−11.28
30	2003-09-12	−0.72	2.34	2003-11-24	−6.88	−9.57
31	2003-09-14	−1.30	2.99	2003-12-04	−1.45	−2.82
32	2003-09-18	−3.12	4.39	2003-12-08	−0.63	−0.34
33	2003-09-20	−2.29	5.07	2003-12-10	−1.15	0.74
34	2003-09-25	−0.29	6.50			
35	2003-09-27	−0.04	6.88			
36	2003-09-30	−0.89	7.17			
37	2003-10-07	−3.02	6.18			
38	2003-10-25	−0.49	−5.78			
39	2003-10-31	−28.32	−10.18			
40	2003-11-07	−0.69	−13.42			
41	2003-11-24	−4.19	−9.57			
42	2003-12-04	−0.08	−2.82			
43	2003-12-08	−0.91	−0.34			
44	2003-12-10	−3.05	0.74			
45	2003-12-14	−1.44	2.50			
46	2003-12-24	−1.38	4.33			
47	2003-12-28	−0.08	4.19

**Table 18:** Catalogues of Forbush Decreases Identified from FTS and Unprocessed CR Data at SNAE station and the associated amplitude of the CR anisotropy (ADV).

Order	Date1	FD1 (%)	ADV (%)	Date2	FD2 (%)	ADV (%)
1	2003-01-04	−1.97	3.53	2003-01-24	−0.20	1.64
2	2003-01-10	−1.98	2.78	2003-01-27	−1.32	1.59
3	2003-01-24	−2.04	1.64	2003-02-02	−1.34	1.64
4	2003-01-27	−4.22	1.59	2003-02-19	−1.43	1.94
5	2003-02-02	−4.31	1.64	2003-03-20	−0.24	2.12
6	2003-02-15	−1.59	1.91	2003-03-31	−1.02	2.02
7	2003-02-19	−4.80	1.94	2003-04-11	−1.58	1.47
8	2003-03-16	−0.78	2.08	2003-04-27	−0.20	0.45
9	2003-03-20	−2.60	2.12	2003-05-03	−0.98	0.21
10	2003-03-31	−4.05	2.02	2003-05-06	−0.28	0.12
11	2003-04-11	−4.64	1.47	2003-05-09	−1.13	0.02
12	2003-04-27	−0.85	0.45	2003-05-19	−0.01	−0.60
13	2003-05-03	−2.17	0.21	2003-05-31	−7.34	−1.84
14	2003-05-06	−0.67	0.12	2003-06-11	−1.79	−4.02
15	2003-05-09	−2.27	0.02	2003-06-16	−2.40	−4.87
16	2003-05-31	−12.83	−1.84	2003-06-23	−4.82	−5.40
17	2003-06-23	−4.23	−5.40	2003-06-28	−2.79	−5.12
18	2003-06-28	−0.47	−5.12	2003-07-04	−1.98	−3.98
19	2003-07-15	−0.43	−0.35	2003-07-08	−1.26	−2.81
20	2003-07-21	−0.25	1.66	2003-07-10	−0.54	−2.14
21	2003-07-27	−6.01	3.07	2003-07-14	−0.54	−0.71
22	2003-08-02	−2.00	3.56	2003-07-27	−1.47	3.07
23	2003-08-05	−1.62	3.45	2003-08-19	−1.15	1.16
24	2003-08-11	−0.53	2.66	2003-10-26	−2.79	−6.60
25	2003-08-19	−3.46	1.16	2003-10-31	−22.04	−10.38
26	2003-09-07	−0.10	2.43	2003-11-07	−7.15	−13.79
27	2003-09-09	−2.26	3.14	2003-11-18	−6.77	−13.33
28	2003-09-12	−1.57	4.35	2003-11-21	−7.70	−12.07
29	2003-09-17	−3.30	6.45	2003-11-23	−7.92	−11.04
30	2003-09-21	−3.21	7.96	2003-11-30	−2.59	−6.64
31	2003-09-27	−1.51	9.32	2003-12-03	−1.60	−4.63
32	2003-09-30	−1.87	9.42	2003-12-08	−0.98	−1.48
33	2003-10-07	−2.29	7.75	2003-12-10	−1.26	−0.37
34	2003-10-31	−33.70	−10.38			
35	2003-11-07	−0.51	−13.79			
36	2003-11-18	−0.21	−13.33			
37	2003-11-23	−4.81	−11.04			
38	2003-12-11	−2.52	0.14			
39	2003-12-23	−0.89	3.82			
40	2003-12-25	−1.21	3.99			
41	2003-12-29	−1.66	4.02			

## 5 Experimental Design, Materials and Methods

### 5.1 Manual identification of Forbush events

CR intensity flux variations are continuously monitored by the ground level neutron monitors (NMs). These variations may be increases or decreases, periodic or sporadic, short- or long-term, and rapid or gradual. FDs are regarded as the most informative events in the CR intensity changes. Unfortunately, NMs do not measure FDs directly. In the NM data, FDs must be distinguished from other variations of similar magnitudes such as the daily and enhanced CR anisotropies. Detecting FDs in CR data involves methods of identification.

Traditionally, the manual method has been used over the past eighty years. This method involves visual inspection of CR data, plotting of some selected segments and calculating the event magnitude. Fig. 10 illustrates the manual technique of detecting an FD event. One of the largest events (FD of 30/5/2003, see Tables 1 and 7) is used to demonstrate the steps involved in the manual approach. This illustration should enable the reader to understand the automated approach presented hereafter.

The method involves culling a certain part of raw CR data and plotting as indicated in panel a of Fig. 10. The plotted portion is first examined to identify the four most important parts of a Forbush event (onset, main phase, minimum point and recovery phase, see Fig. 10). The blue arrow shows a range of onset days that may be visually associated with the event of 30/5/2003 at APTY station. Generally, an FD event includes a decrease lasting a day or more, depending on whether the onset is sudden or gradual, before reaching a minimum. After the minimum the CR flux recovers over some days. The recovery may be rapid or slow.

The plotted event may be discarded if any of the parts described is missing. If the researcher confirms these parts by visual inspection, the experiment is taken to the next stage - calculation of FD event magnitude. This stage also involves several other steps. The researcher chooses the event onset and the minimum point of intensity reduction. While identifying the time of the minimum is usually easy, the time of onset may be more difficult as indicated in panel a.

The next step involves normalization and calculation of the event magnitude. There are two approaches to this, depending on the choice of the normalization baseline. The mean CR variation within the period may be used as the event threshold. On the other hand, the CR count on the day of FD minimum (labeled in panels a and b) may be used as the baseline. Separate equations are used for the two approaches. Equation 1 can be used to estimate event magnitude for the raw data displayed in panel a.

(1) $${\text{FD}}_{\text{amplitude}}\left(\%\right)=\frac{{\text{FD}}_{\text{min}+1}-{\text{FD}}_{\text{min}-1}}{{\text{FD}}_{\text{min}}}\times 100\%,$$

where ${\text{FD}}_{\text{min}}$ represents the CR count value on the day of minimum reduction.

Equation 2 is used to calculate FD magnitude from panel b of Fig. 10.

(2) $${\text{FD}}_{\text{amplitude}}\left(\%\right)=\frac{{\text{FD}}_{\text{onset}}-{\mathrm{mean}}_{I}}{{\mathrm{mean}}_{I}}\times 100\%,$$

where ${\mathrm{mean}}_{I}$ represents the mean value of the CR data for a predefined period.

The FD magnitudes reported in panels a and b are calculated using Eqs. (1) and (2) respectively.

### 5.2 Automated Identification of Forbush Events

Two FD location algorithms are employed here. The first, referred to as R-FD Location Algorithm, is capable of handling raw CR data whereas the second (R-Fourier-FD Location) takes Fourier transformed CR data as its input data. Rather than testing the occurrence of Forbush event using a selected portion of CR data as demonstrated in the manual method, the two codes can take complete CR data for one or more years as its input signal. Panel a of Fig. 11 presents APTY data for the whole of 2003. The large FD of 30/5/2003 plotted in Figure 10 are clearly marked. The largest event of 31/10/2003 is also evident. Other smaller dips (indications of FDs) are also noticeable. The data are used as input signal for the two algorithms. The two methods are briefly described below.

• R-FD Location Algorithm

This algorithm is designed to search for dips/depressions/turning points in the unprocessed CR data. These dips are indications of FDs. It also records the time of the depressions. The search for the dips and the time of occurrence are executed by two subroutines implemented in the code. Normalization and filtering steps (Eq. (2)) similar to those implemented in the manual method are also employed here. The ${\mathrm{mean}}_{I}$here represents the mean value of the CR data for the year 2003.

Using the mean as the normalization baseline, the first subroutine calculates the size of the dips while the second simultaneously records the time of the depressions. The result of normalization and data filtering is displayed in panel b of Fig. 11. The event magnitude and date calculated by the program are presented Table 10. The black signals (labeled Raw CR data) and the associated filled circles in Fig. 2, Fig. 3, Fig. 4, Fig. 5, Fig. 6, Fig. 7, Fig. 8, Fig. 9 are other forms of outputs of the algorithm.

• R-Fourier-FD Location Algorithm

The design of this code is very similar to the R-FD program. All the subroutines included in R-FD Location code are integrated in the R-Fourier-FD code. The major difference lies in the Fourier transformation subroutine which is implemented in the R-Fourier-FD code. The code is developed in [9] and implemented in [6]. We briefly describe the experimental set up.

Either of the data displayed in panels a and b can both serve as input data to the code. Note that the difference in the lies in the normalization and filtering steps. The two datasets are basically considered raw/unprocessed as the the presence of CR anisotropy has not been removed. The input data is then treated as a Fourier series composed of several harmonics. The two major components of interest are the high and low frequency signals. Rather than dealing with several other superimposed signals separately, the raw data is separated into two component signals - the high frequency and low frequency signals. The two signals are presented in panels c and d of Fig. 11. This high frequency signal of the CR data contains the Forbush events. The signal is passed to R-FD location code. The code calculates both the FD magnitude and the time of occurrence. The high frequency signal is labeled FTS (Fourier transformed signal) in Fig. 2, Fig. 3, Fig. 4, Fig. 5, Fig. 6, Fig. 7, Fig. 8, Fig. 9.

The low frequency signal contains both the linear trend (monotonic variation) and the CR anisotropy. In other to remove the linear trend, normalization and filtering steps are further implemented. The resulting signal is displayed in Fig. 2, Fig. 3, Fig. 4, Fig. 5, Fig. 6, Fig. 7, Fig. 8, Fig. 9 as ”Diurnal wave”. A simple coincidence algorithm is used to identify the amplitude of the diurnal anisotropy for each of the FDs detected at the stations. Some of the results are presented in Table 9, Table 10, Table 11.

## Ethics Statement

Not applicable.

## Declaration of Competing Interest

We declare that there is no conflict of interest.

## Appendix A Supplementary Materials

Supplementary Data S1 Supplementary Raw Research Data. This is open data under the CC BY license http://creativecommons.org/licenses/by/4.0/ Supplementary Data S1
